# Supplementary material for: Antibodies from malaria-exposed Malians generally interact additively or synergistically with human vaccine-induced RH5 antibodies
Source: Cell Rep Med. 2021 Jun 21;2(7):100326. doi: 10.1016/j.xcrm.2021.100326 (PMC8324462; doi:10.1016/j.xcrm.2021.100326)
Supplement: Document S1. Figures S1–S5 [file mmc1.pdf]

Cell Reports Medicine, Volume *2*

Supplemental information

Antibodies from malaria-exposed Malians generally interact additively or synergistically with human vaccine-induced RH5 antibodies

Alexandra C. Willcox, Alex S. Huber, Ababacar Diouf, Jordan R. Barrett, Sarah E. Silk, David Pulido, Lloyd D.W. King, Daniel G.W. Alanine, Angela M. Minassian, Mahamadou Diakite, Simon J. Draper, Carole A. Long, and Kazutoyo Miura

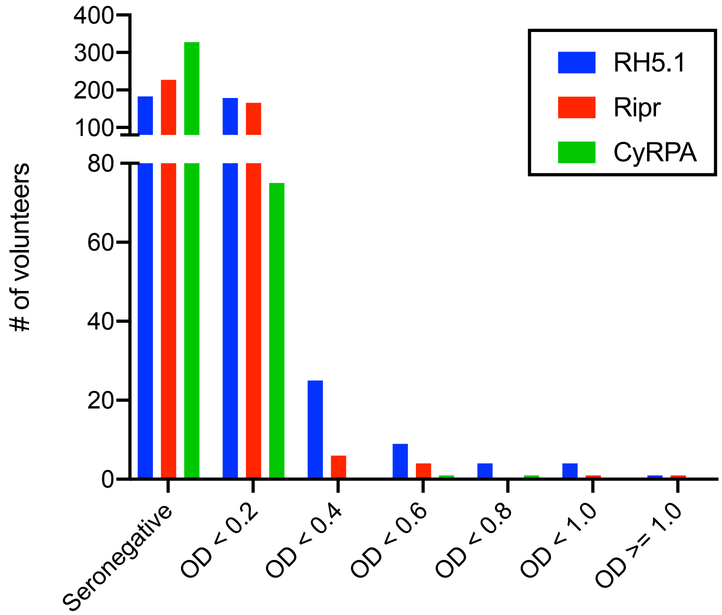

Figure S1: Antibody responses against RH5.1, Ripr and CyRPA. Related to Figure 1. Malian serum samples (n=405) and U.S. malaria-naive sera (n=12) were tested at 1:200 dilutions against the three proteins. After subtraction of background signal (i.e., average O.D. of the 12 U.S. sera), the Malian samples were categorized based on O.D. value.

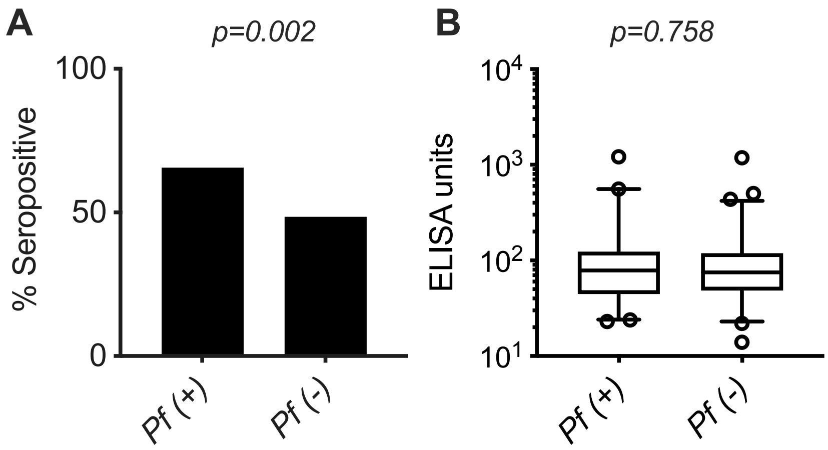

Figure S2: Association between *P. falciparum* infection at the time of sample collection and seropositivity for RH5.1. Related to Figure 1. Out of the 405 volunteers, 391 individuals had *P. falciparum* (Pf) infection status determined by PCR at the time of serum collection; 125 were Pf positive and 266 were Pf negative. (A) The proportion (%) of RH5.1 seropositive participants in each Pf positive or negative category is shown with a Fisher exact p value. (B) The distribution of ELISA units against RH5.1 in seropositive only participants with and without Pf infection (i.e. excluding seronegative participants; n=82 for Pf(+) and n=129 for Pf(-)). A box plot of the data with 95th percentile error bars is shown with the Mann-Whitney p-value.

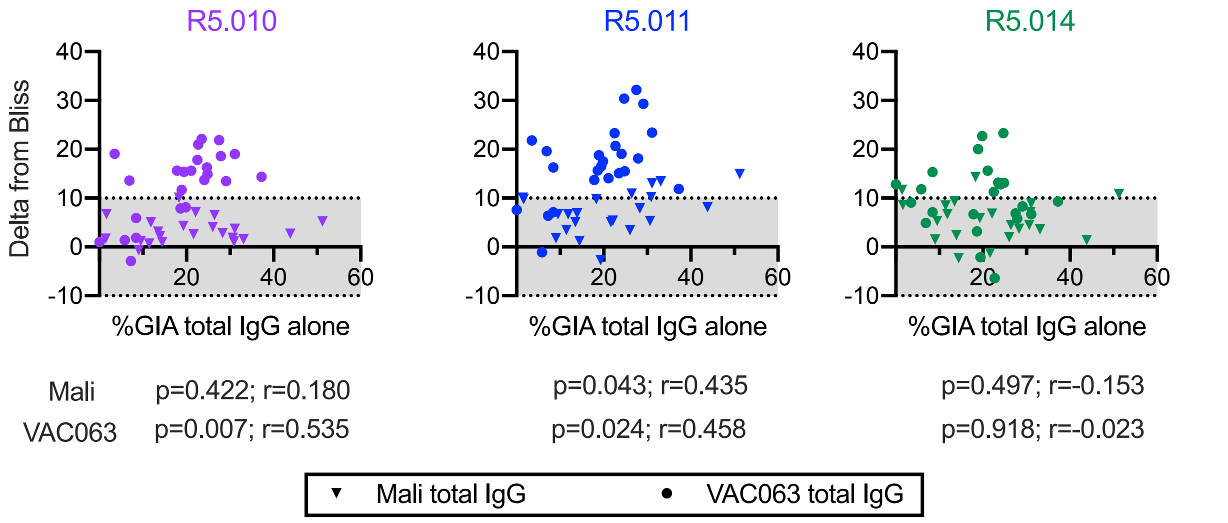

Figure S3: Initial screening data for combination GIA with potentiating RH5 mAbs. Related to Figure 4. Twenty-two Malian individual total IgGs and 24 VAC063 individual total IgGs were independently mixed with the three potentiating mAbs in GIA. The %GIA of total IgG alone is plotted against the Delta from Bliss value for combinations with each RH5 mAb. The Spearman rank test p-value and correlation coefficient r for each group of total IgGs against each mAb is shown. Within 10 Delta from Bliss results (gray areas) were considered additive (no potentiation).

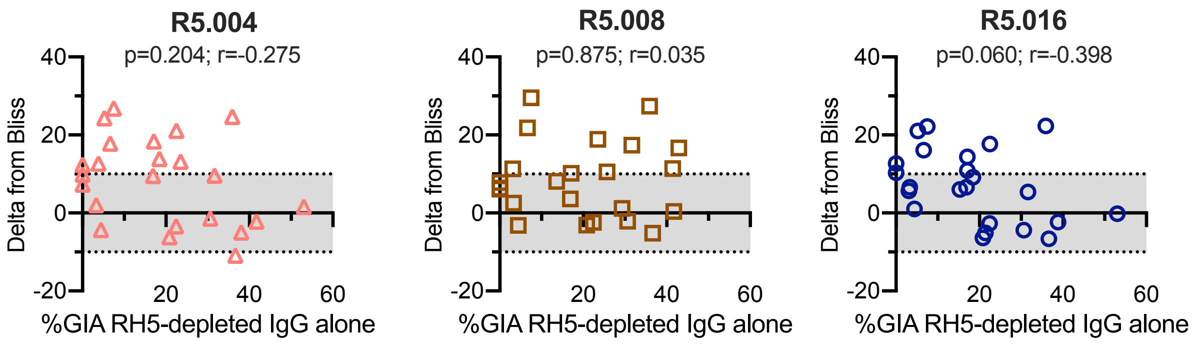

Figure S4: Association between GIA activity of Malian RH5-depleted IgG alone and Delta from Bliss value for combinations with RH5 mAbs. Related to Figure 5. The %GIA of each Malian RH5-depleted IgG alone is plotted against the Delta from Bliss value for combinations with each RH5 mAb, with Spearman rank test p-values and correlation coefficient r. Within 10 Delta from Bliss results (gray areas) were considered additive.

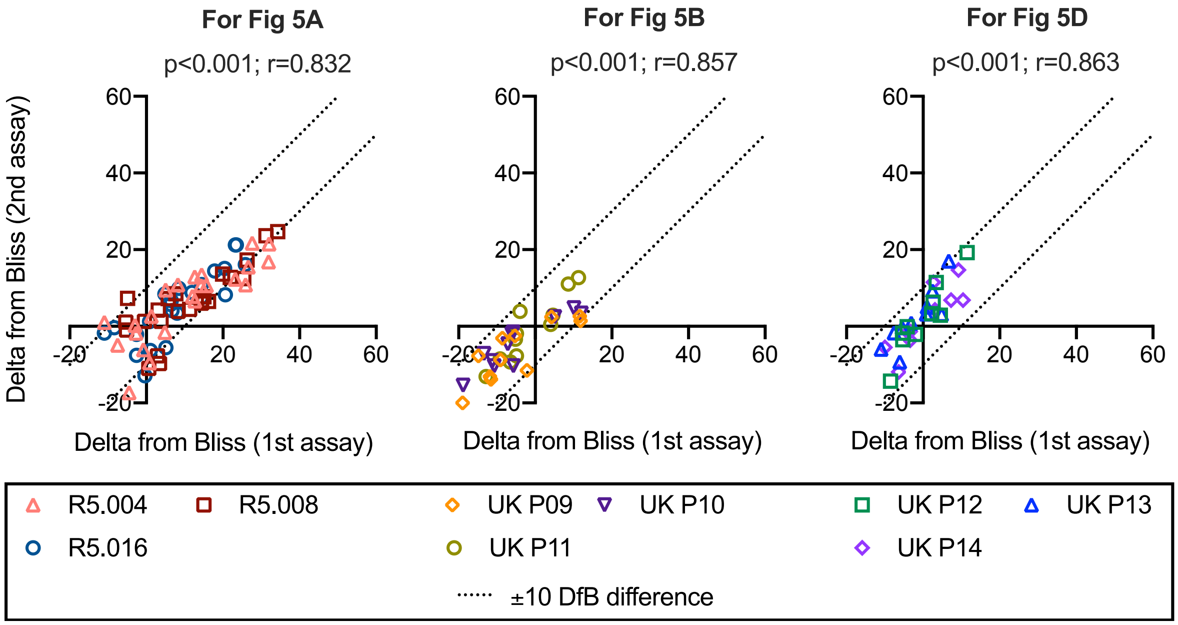

Figure S5: Inter-assay variability in Delta from Bliss value. Related to Figure 5. Independent combinations of Malian RH5-depleted IgGs and three RH5 mAbs (Fig 5A), combinations of Malian RH5-depleted IgGs and VAC063 pooled total IgGs (Fig 5B), or combinations of Malian total IgGs and VAC063 pooled total IgGs (Fig 5D) were assessed in two independent GIAs. The Delta from Bliss (DfB) value determined in the first assay was plotted against that from the second assay. The Pearson test p-value and correlation coefficient r for each data set is shown.
