# Supplementary material for: Antibodies from malaria-exposed Malians generally interact additively or synergistically with human vaccine-induced RH5 antibodies
Source: Cell Rep Med. 2021 Jun 21;2(7):100326. doi: 10.1016/j.xcrm.2021.100326 (PMC8324462; doi:10.1016/j.xcrm.2021.100326)
Supplement: Document S2. Article plus supplemental information [file mmc7.pdf]

Cell Reports Medicine

Article

Antibodies from malaria-exposed Malians generally interact additively or synergistically with human vaccine-induced RH5 antibodies

Graphical abstract

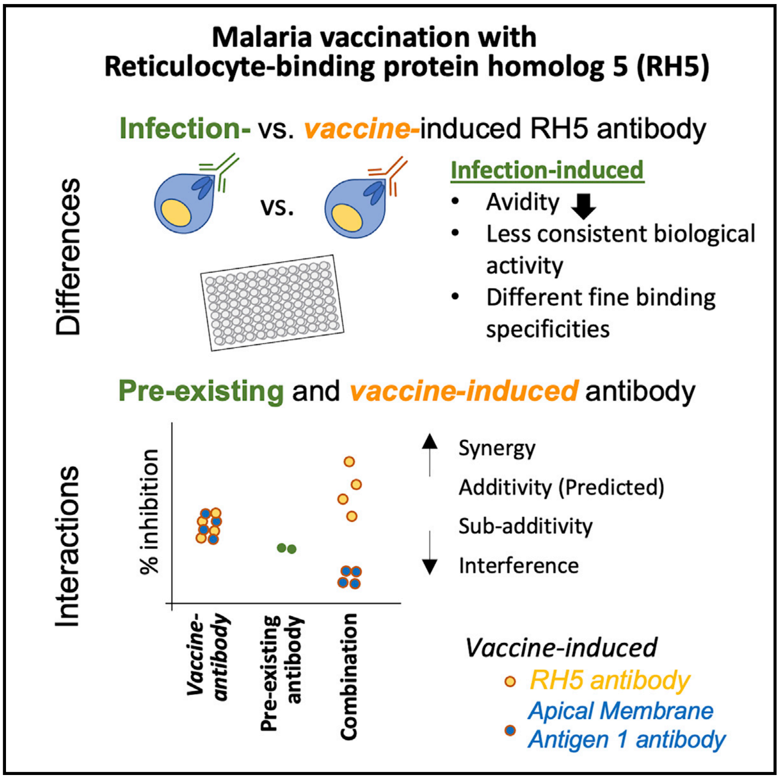

Highlights

- RH5 IgG titers induced by infection are lower than those induced by RH5 vaccination
- Infection- and vaccine-induced RH5 IgGs have different specificity and avidity
- Infection- and vaccine-induced RH5 IgGs interact differently with RH5 mAbs
- Infection-induced IgGs generally do not reduce the activity of vaccine-induced IgGs

Authors

Alexandra C. Willcox, Alex S. Huber, Ababacar Diouf, ..., Simon J. Draper, Carole A. Long, Kazutoyo Miura

Correspondence

kmiura@niaid.nih.gov

In brief

Willcox et al. combine antibodies from malaria-exposed Malians and volunteers who received a reticulocyte-binding protein homolog 5 (RH5) vaccine. In *P. falciparum* growth inhibition assays, infection-induced IgGs generally do not reduce the neutralizing activity of vaccine-induced IgGs. These results suggest that RH5 vaccines will induce effective antibodies in malaria-endemic populations.

Willcox et al., 2021, Cell Reports Medicine 2, 100326
July 20, 2021
https://doi.org/10.1016/j.xcrm.2021.100326

CellPress

Cell Reports Medicine

CellPress OPEN ACCESS

Article

Antibodies from malaria-exposed Malians generally interact additively or synergistically with human vaccine-induced RH5 antibodies

Alexandra C. Willcox,[1] Alex S. Huber,[1] Ababacar Diouf,[1] Jordan R. Barrett,[2] Sarah E. Silk,[2] David Pulido,[2] Lloyd D.W. King,[2] Daniel G.W. Alanine,[2] Angela M. Minassian,[2] Mahamadou Diakite,[3] Simon J. Draper,[2] Carole A. Long,[1] and Kazutoyo Miura[1,4,*]

[1]Laboratory of Malaria and Vector Research, National Institute of Allergy and Infectious Diseases, National Institutes of Health, Rockville, MD 20852, USA

[2]The Jenner Institute, University of Oxford, Old Road Campus Research Building, Oxford OX3 7DQ, United Kingdom

[3]Malaria Research and Training Center, Faculty of Medicine, Pharmacy, and Odontostomatology, University of Sciences, Techniques, and Technologies of Bamako, Bamako 1805, Mali

[4]Lead contact

*Correspondence: kmiura@niaid.nih.gov

https://doi.org/10.1016/j.xcrm.2021.100326

SUMMARY

Reticulocyte-binding protein homolog 5 (RH5) is a leading *Plasmodium falciparum* blood-stage vaccine candidate. Another possible candidate, apical membrane antigen 1 (AMA1), was not efficacious in malaria-endemic populations, likely due to pre-existing antimalarial antibodies that interfered with the activity of vaccine-induced AMA1 antibodies, as judged by *in vitro* growth inhibition assay (GIA). To determine how pre-existing antibodies interact with vaccine-induced RH5 antibodies, we purify total and RH5-specific immunoglobulin Gs (IgGs) from malaria-exposed Malians and malaria-naive RH5 vaccinees. Infection-induced RH5 antibody titers are much lower than those induced by vaccination, and RH5-specific IgGs show differences in the binding site between the two populations. In GIA, Malian polyclonal IgGs show additive or synergistic interactions with RH5 human monoclonal antibodies and overall additive interactions with vaccine-induced polyclonal RH5 IgGs. These results suggest that pre-existing antibodies will interact favorably with vaccine-induced RH5 antibodies, in contrast to AMA1 antibodies. This study supports RH5 vaccine trials in malaria-endemic regions.

INTRODUCTION

Malaria remains one of the biggest threats to global health, with estimates of 228 million clinical cases and 405,000 deaths in 2018.[1] Existing antimalarial control measures, such as insecticide-treated nets, rapid diagnostics, and antimalarial drugs, have reduced malaria cases and deaths dramatically in the last two decades; however, the incidence rate was unchanged between 2014 and 2018.[1] Therefore, it is necessary to supplement existing control measures with new tools, such as vaccines.

Reticulocyte-binding protein homolog 5 (RH5) is one of the leading blood-stage vaccine candidates for *Plasmodium falciparum*, which is the most lethal *Plasmodium* species that causes malaria in humans. RH5 is expressed on merozoites and binds to its specific receptor, basigin, on the surface of erythrocytes.[2] RH5 forms a complex with the RH5-interacting protein (Ripr) and cysteine-rich protective antigen (CyRPA),[3] and the formation of this complex is an essential step during parasite invasion.[4] In addition to supportive evidence from longitudinal cohort studies showing associations between anti-RH5 titer and reduced risk of clinical malaria,[5–8] RH5 vaccination[9] and monoclonal antibody (mAb) inoculation[10] induced protection against *P. falciparum* in an *Aotus* monkey challenge model. Polyclonal and monoclonal antibodies raised against RH5 in animals[11–16] and humans[17,18] have shown biological activity, as judged by *in vitro* growth inhibition assay (GIA). In the two *Aotus* challenge studies, significant positive correlations were observed between *in vitro* GIA activity of antibodies and *in vivo* protective effects.[9,10] Furthermore, a recent phase I/IIa trial, conducted in a malaria-naive population, demonstrated reduced *P. falciparum* blood-stage multiplication rates following RH5.1/AS01$_B$ vaccination[19] and controlled human malaria infection (CHMI). This *in vivo* impact on parasite growth significantly correlated with *in vitro* GIA activity before the challenge.[20]

Apical membrane antigen 1 (AMA1) is one of the best-studied *P. falciparum* blood-stage vaccine candidates, and multiple human phase I trials and two phase IIb efficacy trials have been conducted (reviewed by Miura[21]). Similar to RH5, AMA1 vaccination elicited GIA-active antibodies in animals and humans and induced protection in *Aotus* challenge studies.[21] However, in phase IIb trials conducted in target populations, only minor strain-specific protection[22] or no protection[23] was observed. In contrast to RH5, which has relatively conserved amino acid sequences,[24] AMA1 is a polymorphic protein,[25] which at least

This is an open access article under the CC BY license (http://creativecommons.org/licenses/by/4.0/).

Table 1. Characterization of cohort in Kenieroba, Mali

Age (years)[a]	No. of volunteers	No. of females (%)	*P. falciparum* malaria cases (SD)[b]
<5	75	39 (52)	1.7 (1.5)
5–7	71	39 (55)	2.0 (1.4)
8–10	82	38 (46)	1.1 (1.1)
11–17	85	38 (45)	0.6 (1.0)
>17	92	65 (71)	0.1 (0.4)
Total	405	219 (54)	1.0 (1.3)

[a]Age groups were selected so that each group included approximately equal numbers of volunteers.

[b]A malaria case was defined as an axillary temperature > 37.5°C and asexual parasite density > 5,000/μL. Average (standard deviation, SD) clinical cases per person during June 2013 to May 2014 are shown.

partially explains the failure of AMA1-based vaccines in field trials. However, AMA1 antibodies in immunized Malian adults and children demonstrated lower GIA activity than predicted from their AMA1 antibody titers even for homologous parasite strains.[26] Subsequent studies revealed that a mixture of their non-AMA1 immunoglobulin Gs (IgGs) and AMA1-specific IgGs showed lower GIA activity than the AMA1-specific IgGs tested alone. This effect was termed antibody interference. The detailed mechanism and fine specificity of the interfering IgGs were not identified, but it has been shown that the interference came from malaria-specific IgGs (IgG affinity purified against malaria lysate),[27] and the effect did not simply result from competition for binding to the AMA1 molecule.[28] If antimalarial IgGs induced by natural infection block the activity of other neutralizing vaccine-induced IgGs (e.g., anti-RH5 IgGs) in a target population, this might reduce the efficacy of the vaccine in the field. However, the existence of such an interference effect against antibodies induced by other blood-stage vaccine candidates has not yet been investigated. Given that RH5 is currently the main focus of blood-stage vaccine development, it is important to determine the interaction between RH5 antibodies induced by vaccination and antimalarial antibodies induced by natural infection. Moreover, in contrast to the interference phenomenon, a study reported a subset of potentiating RH5 human mAbs that have no GIA activity on their own but can increase the GIA activity of other neutralizing antibodies by extending the time required for merozoites to invade erythrocytes.[18] This discovery offers an exciting new avenue for increasing the potency of both RH5-based and non-RH5-based vaccines.

In this study, we purified RH5-specific IgGs from malaria-immune Malians, as well as from malaria-naive UK adult vaccinees immunized with a full-length recombinant RH5 protein (RH5.1)[19] formulated in $AS01_B$ adjuvant in a trial designated VAC063, to compare the characteristics of the antibodies. Next, Malian and VAC063 total and RH5-specific IgGs were mixed with potentiating mAbs in GIA to elucidate synergistic interactions. Lastly, Malian RH5-depleted IgGs were mixed with neutralizing human RH5 mAbs and VAC063 polyclonal antibodies (pAbs) to determine the interaction of vaccine-induced antibodies and pre-existing antibodies in GIA. The interactions were generally additive, and a substantial interference effect was not observed, as was seen in the case of AMA1. Thus, this study supports continued trials of RH5-based vaccines in malaria-endemic areas.

RESULTS

Epidemiology of IgG responses against RH5-CyRPA-Ripr complex proteins in Mali

The study cohort consisted of 500 individuals aged 1–65 living in the village of Kenieroba, Mali, an area of high malaria transmission with a rainy season from June to December.[29] Serum samples were collected from 405 volunteers at the peak of the malaria transmission season (October). The 405 participants, divided into five age groups with approximately equal numbers of volunteers, are summarized in Table 1. The average number of clinical malaria cases was 1.0 per person per year, with the 5–7 age group experiencing the highest average of 2.0 (Table 1).

The seropositivity of the 405 Malian serum samples was determined by comparing the ELISA optical density (OD) values against sera from 12 U.S. malaria-naive individuals for each protein or *P. falciparum* FVO parasite lysate (PfFVO). IgG antibodies against PfFVO were undetectable in only 2.7% of all tested sera, underlining the high endemicity of Kenieroba (Figure 1A). By contrast, IgG responses against RH5.1, Ripr, and CyRPA recombinant proteins were detectable in 54.8%, 43.5%, and 19.0% of participants, respectively. The likelihood of a participant having detectable antibodies increased with age for the parasite lysate and all three recombinant proteins (Figure 1A; $p < 0.001$ for all by chi-square test for trend). The OD values against Ripr and CyRPA were too low to investigate further (Figure S1); therefore, quantitative ELISA units (EUs) were determined only against PfFVO and RH5.1 for seropositive participants. Age positively correlated with PfFVO and RH5.1 EUs in participants under the age of 17 ($p < 0.001$ for both), but not over the age of 17 (Figure 1B). There was a weak but significant positive correlation between RH5.1 and PfFVO EUs (Figure 1C; $p < 0.001$). RH5.1 EUs in Malians (only seropositive volunteers) were compared with the peak EUs in volunteers from the VAC063 RH5 vaccine trial (Figure 1D) within the same age range (i.e., over the age of 17). The median RH5.1 EU was approximately 170-fold higher in VAC063 volunteers than in the Malian cohort (15,600 and 91 EU, respectively; $p < 0.001$). *P. falciparum* infection at the time of sample collection was associated with seropositivity against RH5.1, but among seropositive participants, *P. falciparum* infection did not associate with higher EU (Figure S2). To determine the biological activity of Malian antibodies, total IgGs were purified from pooled (n = 23) and individual (n = 24, selected based on available volume) Malian sera and tested in GIA. The total IgGs exhibited activity ranging from 0% to 100% inhibition in GIA (%GIA) at 10 mg/mL, and this functional activity correlated with the PfFVO ($p < 0.001$) and RH5.1 ($p = 0.002$) EUs (Figure 2).

Comparison of RH5-specific IgGs from naturally exposed Malians and vaccinated volunteers

To evaluate the characteristics of naturally acquired RH5-specific IgGs, we purified RH5-specific IgGs from each of the 23 Malian pooled total IgGs. Eight of the pools with relatively high RH5.1 EU yielded RH5-specific IgGs of sufficient volume and concentration for further experiments (open symbols in Figure 2).

CellPress
OPEN ACCESS

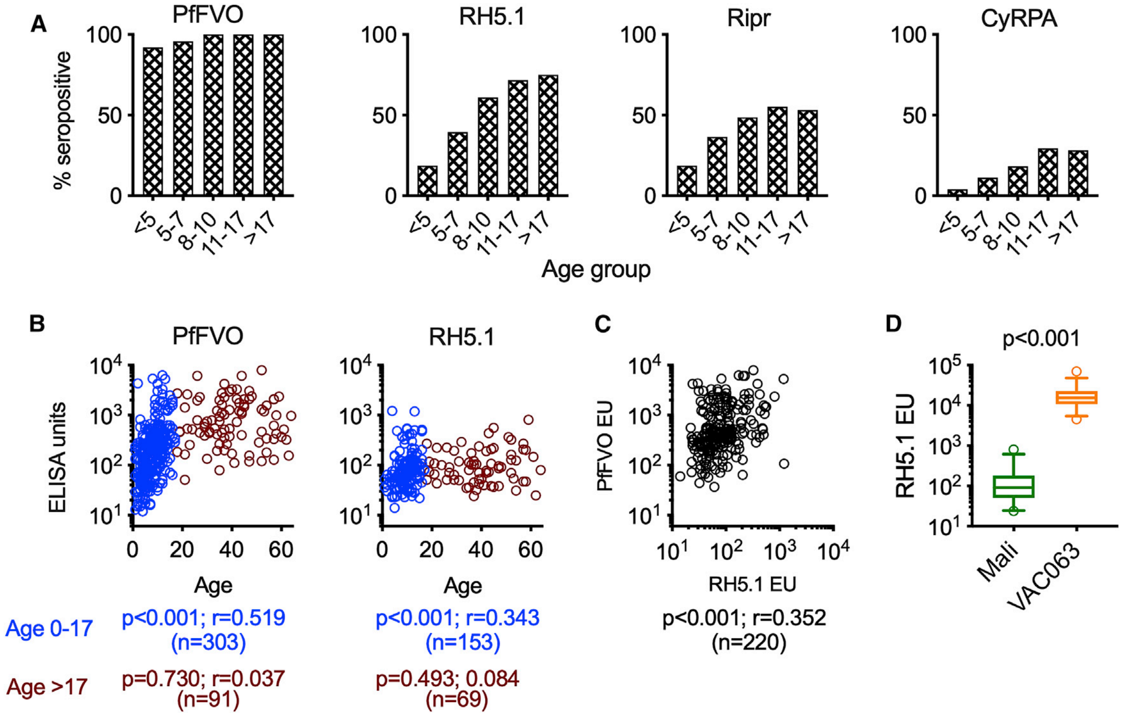

Figure 1. RH5 IgG antibody responses in Malians

(A) Proportion of participants in each age group with a detectable IgG antibody level against *P. falciparum* FVO strain blood-stage parasite extract (PfFVO) and recombinant proteins RH5.1, Ripr, and CyRPA.

(B) Correlation between age and ELISA units (EUs) for participants with detectable antibodies against PfFVO and RH5.1. Spearman rank p values, correlation coefficient r, and number of seropositive volunteers are shown for age groups 0–17 and >17.

(C) Correlation between RH5.1 and PfFVO EU values among seropositive volunteers. Spearman rank p and correlation coefficient r are shown with the number of individuals n.

(D) For all RH5 seropositive Malian adults (over the age of 17), the antibody levels are compared with the peak titers in vaccinees from the UK RH5 trial VAC063. Boxplots (25/50/75 percentiles) with 95 percentile data (error bars) are shown with the Mann-Whitney p value (n = 69 for Mali and n = 69 for VAC063 samples). See also Figures S1 and S2.

For comparison, eight total IgG pools were made from the VAC063 vaccine trial, and RH5-specific IgG purification was performed similarly.

Serial dilutions of the five highest-titer VAC063 RH5-specific IgGs were tested in GIA, and there was a sigmoid correlation between RH5.1 EU and %GIA, as shown in a previous human trial (VAC057)[17] (Figure 3A). The activity of the VAC063 RH5-specific IgGs was then compared with the activity of the five total IgG pools from which they were purified. On a plot of EU versus %GIA, the overlay of VAC063 RH5-specific and total IgGs suggests that the affinity purification process did not change the functional activity of the antibodies (Figure 3A). The five highest-titer Malian RH5-specific IgGs were tested only at a single high concentration in GIA due to limited volumes (Figure 3A). The highest-titer Malian RH5-specific IgG (3,738 EU) failed to show GIA activity (0%GIA), although both total and RH5-specific VAC063 IgGs tested at the same EU showed ~30%GIA. However, the second-highest-titer Malian RH5-specific IgG (2,856 EU) demonstrated 21%GIA, which was similar to the VAC063 samples. The other 3 Malian RH5-specific IgGs were tested with <800 EU and showed <10%GIA.

We next compared all eight Malian and eight VAC063 RH5-specific IgGs for differences in binding to the RH5 protein at equivalent EUs against RH5.1. The RH5.1 protein was used in previous ELISAs (Figures 1 and 2), to capture RH5-specific IgGs in affinity purification, and for vaccination in the VAC063 trial. The avidity of the Malian RH5-specific IgGs was lower than that of those from the VAC063 trial ($p < 0.001$; Figure 3B). Next, two truncated versions of the RH5 protein were used in ELISA: RH5ΔNL[18] (RH5 lacking the N terminus and disordered loop) and RH5Nt[30] (N terminus of RH5). When UK volunteers were previously immunized with full-length RH5 using a chimpanzee adenovirus serotype 63 and modified vaccinia virus Ankara (ChAd63-MVA) vaccination platform (VAC057 trial), most antibodies recognized RH5ΔNL, but only a minor portion reacted with RH5Nt.[17] In the current study, the VAC063 RH5-specific IgGs bound almost equally to RH5ΔNL and RH5Nt (Figures 3C and 3D). Compared with the Malian RH5-specific IgGs, the VAC063 RH5-specific IgGs bound more to RH5ΔNL ($p < 0.001$; Figure 3C), whereas the Malian RH5-specific IgGs showed more binding to RH5Nt, although there was large variation in the Malian samples ($p = 0.050$; Figure 3D). The VAC063 RH5-specific IgGs also showed more binding to the reconstituted recombinant RH5-CyRPA-Ripr complex (referred to hereafter as the RH5 complex) than did the Malian RH5-specific IgGs ($p < 0.001$; Figure 3E).

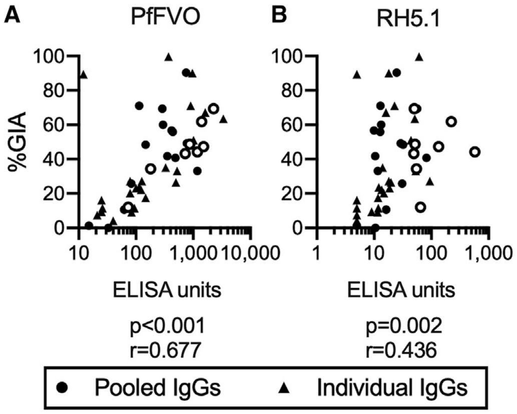

Figure 2. Functional antimalarial activity of Malian total IgGs correlated with PfFVO and RH5 ELISA units

ELISA units against PfFVO (A) and RH5.1 (B) in purified total IgGs from 47 Malian samples (23 serum pools and 24 individual sera) correlated with the percentage of inhibition measured by *in vitro P. falciparum* growth inhibition assay (%GIA) at 10 mg/mL. Spearman r and p values are shown. RH5-specific IgGs were collected from the eight pooled total IgGs (open circles) with adequate volume and relatively high RH5.1 EU for the next experiment, seen in Figure 3.

Potentiating RH5 mAbs boosted GIA activity of VAC063 RH5-specific and total IgGs, but not Malian total IgGs

R5.010, R5.011, and R5.014 are called potentiating mAbs because although they do not exhibit GIA activity on their own, but they have been shown to increase the activity of other antibodies against several merozoite proteins.[18] The potentiating mAbs were individually mixed with the three highest-titer Malian RH5-specific IgGs and with the five highest-titer VAC063 RH5-specific IgGs. The Malian RH5-specific IgGs were tested at the same concentration as used in Figure 3A, and the VAC063 RH5-specific IgGs were diluted to a fixed RH5.1 EU, at which concentration all VAC063 IgGs were expected to give 60% GIA–70%GIA. The original %GIA results are shown in Table S1. The difference between the predicted %GIA (based on the formula for Bliss additivity) and the observed %GIA in a mixture of two antibodies is denoted as Delta from Bliss (DfB; details can be found in the STAR Methods section). Because the potentiating mAbs by themselves do not have activity, in these specific antibody combinations, the predicted %GIA was the same as the %GIA of the RH5-specific IgGs alone, and DfB was calculated as the difference between the %GIA in combination and the %GIA of the RH5-specific IgGs alone. When the synergistic (potentiating) interaction was defined as DfB (from a single assay) being ≥ 10, the R5.010 and R5.011 mAbs showed a potentiating effect with all five VAC063 RH5-specific IgGs and with two of three Malian RH5-specific IgGs. However, R5.014 demonstrated potentiation with two of five VAC063 and one of three Malian RH5-specific IgG samples (Figure 4A; Table S1).

The three potentiating mAbs were also tested in combination GIAs with Malian (n = 22) and VAC063 (n = 24) individual total IgGs, and the original %GIA results are shown in Table S2. Of the 22 Malian individual total IgGs tested, one (5%), six (27%), and three (14%) IgGs showed DfB $\geq$ 10, whereas 17 (71%), 20 (83%), and 11 (46%) of 24 VAC063 individual total IgGs showed DfB $\geq$ 10 by R5.010, R5.011, and R5.014, respectively, in the first single experiment (Figure S3). There were positive correlations between %GIA of the total IgG sample by itself and DfB for some combinations, but not for others (Figure S3). To confirm the results, GIA combinations that showed potentiation in this initial screening were repeated twice with different red blood cells (Figure 4B), because we have observed that the activity (i.e., %GIA) of RH5 antibodies can be affected by the batch of red blood cells used in the assay. Because limited amounts of RH5-specific IgGs were available, the combination GIA for Figure 4A was performed only once. However, all other GIA experiments were performed in two independent assays to minimize the batch effect. Unless otherwise described, the %GIA or DfB values reported hereafter are average values from two assays. We considered an additive interaction (i.e., the two antibodies worked independently) as average DfB < ±10, a synergistic (potentiating) interaction as DfB $\geq$ 10, and an antagonistic interaction as DfB ≤ -10. The ±10 threshold was determined by analyzing variations in the 190 paired combination GIAs performed in this study (i.e., instances in which the same antibody combination was tested in two independent assays). Among these 190 paired GIAs, the absolute values of the 25th, 50th (median), and 75th percentile differences in DfB values between the two assays were 2.2, 5.1, and 9.7, respectively (if there was no error in the assay, the difference in DfB between the two assays would be zero). For Malian individual total IgGs, only zero, three and one IgG showed consistent potentiating interactions (i.e., the average DfB values were $\geq$10) by R5.010, R5.011, and R5.014, respectively, in the confirmatory GIAs. However, 15, 20, and 11 VAC063 individual total IgGs showed potentiation, respectively (Figure 4B). Altogether, these results indicate that the potentiating mAbs had synergistic effects with RH5 antibodies in the VAC063 samples as expected, but had no or minimal impact on overall GIA activity induced by natural malaria infections in the Malian samples.

Malian polyclonal IgGs interacted additively or synergistically with neutralizing RH5 mAbs and mostly additively with VAC063 total IgGs

In a previous study, mixing Malian AMA1-depleted IgGs with neutralizing AMA1 IgGs from AMA1-vaccinated volunteers resulted in an interference effect in GIAs (i.e., the mixture resulted in lower %GIA than either antibody alone).[28] To explore whether a similar phenomenon exists for RH5, the effect of adding neutralizing vaccine-induced RH5 human mAbs to Malian IgGs depleted of RH5-binding antibodies was assessed in combination GIA. The combination GIAs were performed using RH5-depleted Malian IgGs, instead of Malian total IgGs, to perform a fair comparison with previous studies that used AMA1-depleted IgGs in similar experiments.[27,28] The neutralizing mAbs (R5.004, R5.008, and R5.016) had GIA activity by themselves.[18] Two independent assays were performed, and the average DfB was calculated for each combination (the original %GIA value for each test condition in each assay can be found in Table S3). Of 23 Malian RH5-depleted IgGs tested, ten, ten, and nine samples showed synergistic effects (i.e., DfB $\geq$ 10) with R5.004, R5.008, and R5.016, respectively. However, only

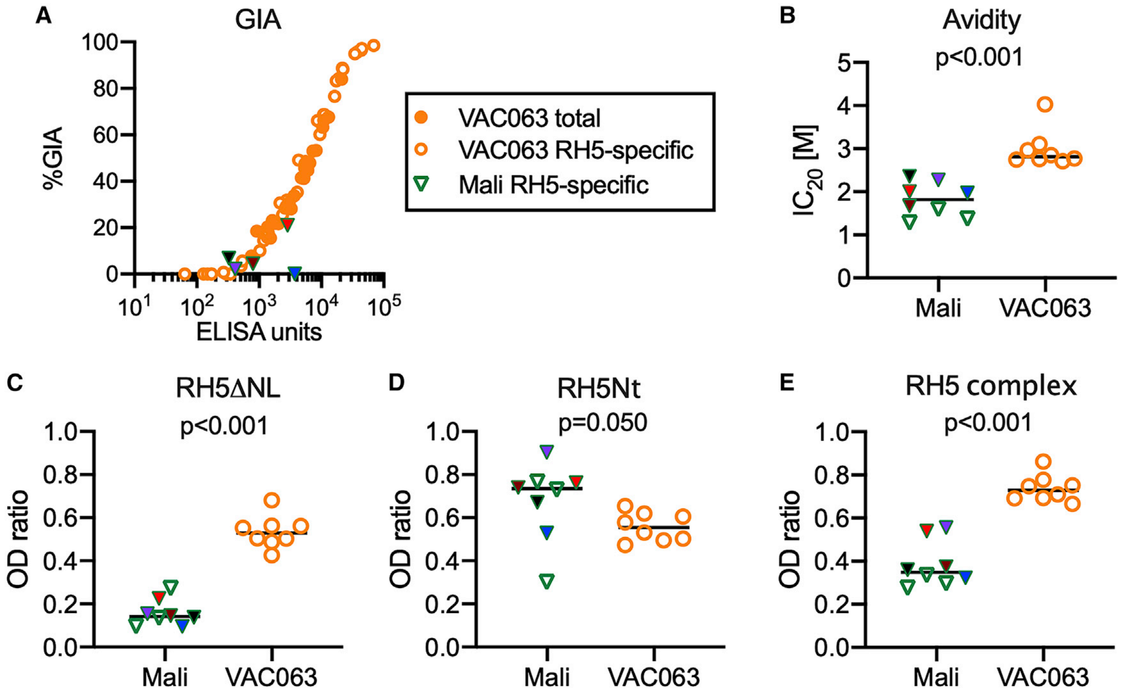

Figure 3. Comparison of RH5-specific IgGs from naturally exposed Malians and vaccinated volunteers

Eight RH5-specific IgGs were collected by RH5 affinity purification from the Malian cohort and from the VAC063 trial.

(A) RH5.1 ELISA units are plotted against %GIA for the five highest-titer RH5-specific IgGs from Malians and both total (n = 5) and RH5-specific (n = 5) IgGs from the VAC063 trial. Each VAC063 total and RH5-specific IgG was tested at multiple dilutions.

(B–E) ELISA was used to measure (B) avidity of the RH5-specific IgGs to recombinant full-length RH5 (RH5.1), (C) binding to a truncated version of RH5, lacking the N terminus and disordered loop (RH5ΔNL), (D) binding to recombinant RH5 N terminus (RH5Nt), and (E) binding to recombinant RH5 complex. For the avidity test, the molar concentration of urea needed to reduce antibody binding by 20% (IC_{20} [M]) is shown.

In (C)–(E), each RH5-specific IgG (either VAC063 or Malian samples) was diluted to target an OD of approximately 1.0 against RH5.1 and the results are expressed as the OD ratio between the protein of interest and the RH5.1 for each sample. For (B)–(E), bars represent the medians, and Mann-Whitney p values are reported. The five highest-titer Malian RH5-specific IgGs, which were used for both GIA (A) and ELISA (B–E), are color coded to facilitate comparison of results in different panels.

one Malian IgG sample mixed with R5.004 showed an antagonistic effect (DfB was −10.9). This mixture condition demonstrated the same %GIA as the activity of R5.004 alone: R5.004 alone had 71%GIA, the Malian RH5-depleted IgG (FT03) alone had 37%GIA, and the mixture had 71%GIA (Table S3). Therefore, this interaction was subadditive (i.e., the mixture of two IgGs demonstrated the same or slightly higher %GIA than either IgG tested alone; see STAR Methods for details). The DfB values of the same Malian RH5-depleted IgG in combination with the three RH5 mAbs correlated significantly (Figure 5A; Spearman rank coefficient ≥ 0.61 with $p \leq 0.002$ for all three paired tests, i.e., R5.004 versus R5.008, R5.008 versus R5.016, and R5.004 versus R5.016). In other words, Malian RH5-depleted IgGs that had higher DfB with one mAb generally also demonstrated higher DfB with the other two mAbs. The DfB value did not correlate with the GIA activity of the Malian RH5-depleted IgG alone (Figure S4; $p > 0.060$ for the three mAbs).

We next explored the effect of combining RH5 polyclonal antibodies (i.e., VAC063 total IgGs) with 11 selected Malian RH5-depleted IgGs (indicated with arrows in Figure 5A), which represented high (n = 4), middle (n = 3), and low (n = 4) DfB samples in experiments with the neutralizing RH5 mAbs. For this combination GIA, three VAC063 pooled total IgG samples were prepared (UK P09, P10, and P11). All combinations of the 11 Malian RH5-depleted IgGs and VAC063 pooled total IgGs were evaluated in two independent assays (the original %GIA values in each assay are in Table S4). In contrast to the combinations with the neutralizing mAbs, DfB values with the VAC063 pooled total IgGs were generally lower (Figure 5B). although four of the Malian RH5-depleted IgGs tested in this combination experiment had shown synergistic effects with the neutralizing RH5 mAbs (Figure 5A), only one Malian RH5-depleted IgG showed a slightly synergistic effect with one of the three VAC063 pooled total IgGs tested (DfB was 12 for the mixture of FT15 and VAC063 P11; Figure 5B). However, four, four, and one Malian RH5-depleted IgGs showed antagonistic effects with VAC063 P09, P10, and P11, respectively. Among the antagonistic interactions, most of them were subadditive, with one exception. One Malian RH5-depleted IgG (FT22) showed 45%GIA by itself, and the mixtures of FT22 with the P09, P10, and P11 IgGs showed 36%GIA, 36%GIA, and 43%GIA, respectively, whereas all three VAC063 pooled total IgGs displayed 15%GIA–29%GIA by themselves. Similar to the combination GIA with neutralizing RH5 mAbs (Figure 5A), the levels of DfB across combinations

Cell Reports Medicine
Article

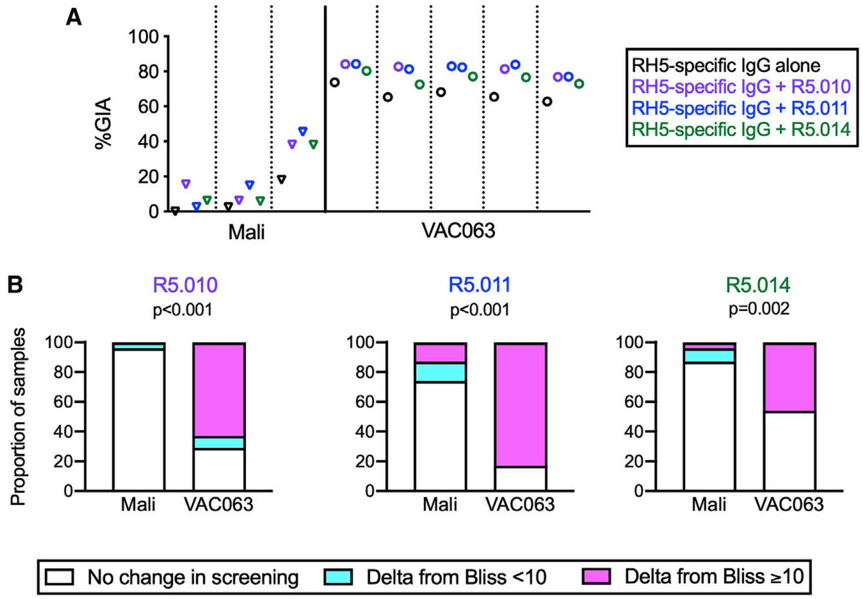

Figure 4. Potentiating RH5 mAbs boosted GIA activity of VAC063 RH5-specific and total IgGs, but not Malian total IgGs

(A) Three potentiating mAbs (R5.010, R5.011, and R5.014) were individually mixed with RH5-specific IgGs from malaria-exposed Malians and vaccinated volunteers in the VAC063 trial. GIA activity is plotted for each RH5-specific IgG on its own (black) and with R5.010 (purple), R5.011 (blue), and R5.014 (green).

(B) 22 Malian individual total IgGs and 24 VAC063 individual total IgGs were independently mixed with the three potentiating mAbs in screening GIA (Figure S3). For the combinations that showed synergy (potentiation; Delta from Bliss [DfB] ≥ 10) in the first screening assay, the GIA was repeated twice, and the average results are presented as a binary (DfB <10 or ≥10). "No change in screening" means a total IgG did not show a potentiating effect in the screening. The proportion of samples that showed synergistic interactions (i.e., DfB ≥10) in the confirmatory GIA was compared between Malian and VAC063 individual total IgGs, and a p value for Fisher's exact test is shown for each mAb.

See also Figure S3 and Tables S1 and S2.

using the three VAC063 pooled total IgGs were correlated (Figure 5B; Spearman rank coefficients ≥ 0.92 with $p < 0.001$ for all three paired tests). There was significant correlation between the median DfB values in combinations with the three mAbs and the median values in combinations with the three VAC063 pooled polyclonal total IgGs (Figure 5C; Spearman rank coefficient = 0.68, p = 0.025), although the actual DfB values were lower in assays with the VAC063 pooled total IgGs.

Finally, combination GIAs were performed using 10 individual Malian total IgGs (without RH5 IgG depletion) that were randomly selected from various age groups and both genders (from 4 to 57 years old, median of 15 years; 5 females and 5 males) and another 3 pools of VAC063 total IgGs in two independent assays (Figure 5D, with the original %GIA values in Table S5). Similar to the combination GIAs with RH5-depleted IgGs, most (8 of 10) of the Malian total IgGs showed additive interactions with all 3 VAC063 pooled IgGs, and the DfB values for combinations with the three VAC063 pooled total IgGs were correlated (Spearman rank coefficients ≥ 0.89 with $p < 0.001$ for all three paired tests). One Malian total IgG (To09) had synergistic effects with all 3 VAC063 IgGs (DfB of 15.4, 11.9, and 11.9 with UK P12, P13, and P14, respectively), and another Malian total IgG (To03) showed an interference interaction with one of the three VAC063 pooled IgGs (DfB of −11.4 for UK P12; To03 alone, UK P12 alone, and the combination demonstrated 13%GIA, 30%GIA, and 28%GIA, respectively). The same To03 IgG had DfB values of −7.7 and −9.1 with UK P13 and P14, respectively (considered additive interactions by our definition).

DISCUSSION

We observed a sharp contrast in the functional activity of infection-induced RH5- and AMA1-specific IgGs, as well as in how vaccine-induced RH5 and AMA1 antibodies interact with pre-existing antimalarial IgGs in individuals living in an endemic area. In the case of AMA1, infection-induced AMA1-specific IgGs from Malians showed the same GIA activity per EU as AMA1 IgGs induced by AMA1 vaccination of malaria-naive adults.[27,28] However, RH5-specific IgGs from Malians did not consistently show the same GIA activity per EU as RH5 IgGs induced by RH5 vaccination of malaria-naive adults. In addition, when 14 AMA1-depleted IgGs from Malians were incubated with vaccine-induced AMA1 antibodies, all mixtures showed lower parasite inhibition than the vaccine-induced AMA1 antibody alone (a phenomenon termed interference).[27,28] In contrast, when Malian RH5-depleted IgGs were mixed with VAC063 total IgGs, of 33 conditions tested (11 RH5-depleted Malian IgGs with 3 VAC063 IgGs), the results were as follows: one combination showed a synergistic effect, 23 conditions were additive, 6 combinations were subadditive, and three combinations showed interference. The interference interactions were observed for a single Malian FT22 IgG; the Malian FT22 IgG alone showed 45%GIA, and the combinations with VAC063 P09, P10, and P11 IgGs showed 36%GIA, 36%GIA, and 43%GIA, respectively. Because the difference between 45%GIA and 36%GIA is debatable (within a 10%GIA difference) considering the error of assay, it is unclear whether there was a true interference interaction in these combinations. Similarly, when combinations of Malian total IgGs and VAC063 total IgGs were examined, of 30 conditions (10 Malian total IgGs with 3 VAC063 IgGs), 3, 26, 0, and 1 combination showed synergistic, additive, subadditive, and interference interactions, respectively (in the final combination, there was only a 2%GIA difference between the mixture and the VAC063 IgG tested alone). Assuming *in vitro* GIA activity correlates with *in vivo* outcome in malaria-exposed populations, as has been shown in an *Aotus* monkey model[9,10] and CHMI in the VAC063 trial,[20] the lack of interference is promising for RH5 vaccine prospects and encourages further investigation of

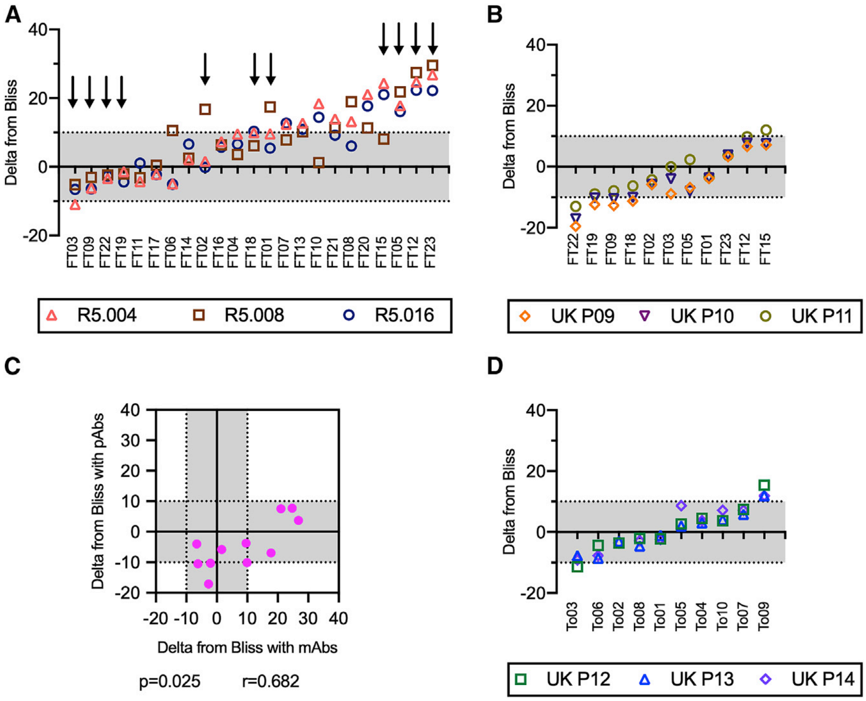

Figure 5. Malian RH5 polyclonal IgGs interacted additively or synergistically with neutralizing RH5 mAbs and mostly additively with VAC063 total IgGs

(A) Independent combinations of 23 Malian RH5-depleted IgGs and three neutralizing RH5 mAbs were assessed in two independent GIAs. The average DfB from the two assays (y axis) was calculated for each Malian RH5-depleted IgG (different columns) mixed with each mAb (different symbols). The Malian RH5-depleted IgGs are sorted by ascending DfB value (median from combinations with the three mAbs). The arrows indicate the RH5-depleted IgG samples tested in the mixture experiments with VAC063 pooled total IgGs in (B).

(B) Independent combinations of 11 Malian RH5-depleted IgGs and three VAC063 pooled total IgGs were assessed in two independent GIAs. The average DfB value from the two assays (y axis) was calculated for each Malian RH5-depleted IgG (different columns) in combination with each VAC063 pooled total IgG (different symbols). The Malian RH5-depleted IgGs were sorted by ascending DfB value (median from combinations with the three VAC063 pooled total IgGs).

(C) Median DfB values from combinations with the three neutralizing mAbs (x axis, mAbs) and the three VAC063 pooled polyclonal total IgGs (y axis, pAbs) were calculated for each Malian RH5-depleted IgG. Each point represents a single Malian RH5-depleted IgG, and a Spearman rank test p value and correlation coefficient r are shown.

(D) Independent combinations of 10 Malian individual total IgGs and three VAC063 pooled total IgGs were assessed in two independent GIAs. The data are presented as in (B). Data points within ±10 of DfB values (gray areas) were considered additive.

See also Figures S4 and S5, and Tables S3, S4, and S5.

RH5 vaccines in endemic areas. Indeed, results from the first phase Ib trial of an RH5-based vaccine in Tanzanian adults, children, and infants are awaited and will provide insight (ClinicalTrials.gov: NCT03435874).

In this study, we screened serum samples collected from 405 Malians at the peak of the malaria transmission season for reactivity against RH5 complex proteins in hopes of generating several RH5-, Ripr-, and CyRPA-specific IgG pools for experiments. However, antibody levels were too low to isolate Ripr- and CyRPA-specific IgGs. Similar to RH5,[6,11,31,32] antibody titers to Ripr[6] and CyRPA[32–34] were previously reported to be low compared with other blood-stage antigens. Therefore, the low titers observed in this study are not surprising, but this study again highlights difficulties in studying infection-induced RH5-, Ripr-, and CyRPA-specific IgGs unless a much larger volume of serum is collected. When humans were immunized with a recombinant RH5 protein, vaccinated individuals demonstrated ~170-fold higher median EU than malaria-exposed individuals, indicating that the RH5 antigen is immunogenic in humans when properly presented (VAC057[17] and this study).

Human RH5-specific IgGs from Senegalese[35] and Malians (in a different cohort from this study)[5] have shown GIA activity in previous studies, but only one RH5-specific IgG pool was examined in each study. Therefore, it was not clear whether there is any difference in the characteristics of different RH5-specific IgGs. In this study, multiple RH5-specific IgG pools from individuals who were naturally infected (Malian IgGs) or vaccinated (VAC063 IgGs) were evaluated and directly compared. Of the two Malian RH5-specific IgGs (the blue and red filled triangles in Figure 3) that could be tested with >1,000 EU in GIA (a level expected to give greater than a background level of %GIA based on the VAC063 total and RH5-specific IgGs' activity), one IgG showed no GIA activity (blue triangle) and the other showed a predicted level of %GIA (red triangle). In general, the Malian RH5-specific IgGs had lower avidity and bound less to RH5ΔNL (RH5 lacking the N terminus and disordered loop) and the RH5 complex but more to RH5Nt (the N terminus of RH5). A previous study in rabbits and humans conducted with full-length RH5 vaccines indicated major GIA epitopes might exist within the RH5-ΔNL region.[17,18] In line with these results, the highest-titer Malian RH5-specific IgG had an undetectable level of binding to RH5-ΔNL and failed to show GIA activity at the expected concentration. In contrast, a higher level of RH5ΔNL binding was exhibited by the GIA-active second-highest-titer Malian IgG. However, when mice and rabbits were immunized with the N terminus of RH5, they produced GIA-active antibodies.[30,36] Concerning binding to the RH5 complex, it is theoretically plausible that RH5 antibodies that bind sites exposed on the RH5 complex may have a longer time to inhibit the invasion process than RH5 antibodies that only recognize epitopes on RH5 that are covered by CyRPA in the complex; however, conflicting results have been published in this regard.[16,24,37] Because only two Malian RH5-specific IgGs could be tested at a high-enough concentration, and the five VAC063 RH5-specific IgGs with strong GIA

activity recognized RH5ΔNL, RH5Nt, and RH5 complex proteins relatively homogeneously, it is still an open question whether the ELISA results on binding characteristics can help explain the lack of GIA activity observed in the highest titer Malian RH5-specific IgG.

Combination GIAs with RH5 antibodies (either mAbs or pAbs) have been performed in many studies,[13,15,16,34,38–40] but the terminology of additive and synergistic needs to be interpreted carefully. Bliss's and/or Loewe's definitions of additivity have been used in many studies,[13,15,34,38,40] whereas other studies used neither definition.[16,39] Bliss and Loewe additivity (and synergy) have different definitions, and both have advantages and disadvantages (a detailed mathematical discussion can be found elsewhere[41]). Bliss additivity can be determined by testing a minimum of three experimental conditions (e.g., antibody A alone at one concentration, antibody B alone at one concentration, and a mixture of the two), but this definition carries a risk of self-synergy or self-antagonism (as described in detail previously[41]). Loewe's definition avoids the self-synergy issue. However, to determine Loewe additivity/synergy, multiple combination GIAs are required for each antibody mixture, because each of the two antibodies must be tested at multiple concentrations.[41] Because limited amounts of human IgGs (especially RH5-specific IgGs) were available in this study, we designed our study to determine the interactions based on Bliss's definition.

The recent discovery of a subset of antibodies that potentiate the activity of neutralizing merozoite antibodies opens up the possibility of increasing the protection conferred by a vaccine. These non-neutralizing, potentiating RH5 mAbs boosted the activity not only of neutralizing RH5 antibodies but also of pAbs from rabbits and rats immunized with other merozoite proteins, including RH4, CyRPA, Ripr, and AMA1, but not MSP1.[18] In this study, the activity of VAC063 RH5-specific IgGs was generally boosted by the potentiating mAbs, and most VAC063 individual total IgGs showed a synergistic effect in combination with the potentiating mAbs. However, the effect was less profound and uniform among the Malian samples. The potentiating mAbs improved the activity of the one Malian RH5-specific IgG pool with baseline activity of ~20%GIA (this finding was reproduced in a biological replicate), but the results with the other two Malian RH5-specific IgGs are more difficult to interpret, because their baseline GIA activity was negligible. For Malian individual total IgGs, only a minor proportion of samples showed synergistic effects. Overall, these results suggest that potentiating mAbs boost vaccine-induced anti-RH5 neutralizing activity but that the effect is negligible in non-vaccinated, malaria-exposed populations with low anti-RH5 antibody titers (such as this Malian population). Together with results from the rodent study,[18] it is possible that the Malian total IgGs contain more MSP1-like IgGs than RH5-like IgGs. An interesting question for future studies is whether the vaccine-induced RH5 antibody activity in the target population could be improved by potentiating antibodies.

In exploring the interaction between vaccine-induced and infection-induced antibodies, we opted to first combine Malian RH5-depleted IgGs with RH5 mAbs from vaccinated individuals and then proceeded to choose a subset of these Malian IgGs to combine with pooled total IgGs from the VAC063 trial. By taking this approach, we discovered several important associations. Not only did the DfB values of a Malian IgG in combination with one of the RH5 mAbs (or polyclonal IgG pool) correlate with values from the same Malian IgG in combination with the other two mAbs (or other polyclonal IgG pools), but the results from the mAb combinations were also significantly correlated with the values from the pAb experiments. Therefore, in future studies (especially with limited total IgG availability), it may be useful to perform a screening approach in which the mAb combinations are performed first and then more combinations with polyclonal antibodies can be performed for selected samples. When Malian IgGs were tested with RH5 mAbs, the combinations showed overall higher DfB than the combinations with RH5 pAbs. The difference could be explained, at least partly, by epitope recognition. The three mAbs bind near the RH5 diamond tip,[18] whereas the pAbs reacted with both RH5ΔNL and RH5Nt proteins. If this is true, a new RH5 protein, designed to immuno-focus on the diamond tip, could elicit more desirable antibodies.

We revealed that functionality and fine specificity differed between vaccine-induced (VAC063) and infection-induced (Malian) RH5 antibodies. Non-neutralizing, potentiating mAbs boosted the activity of vaccine-induced RH5 antibodies more so than naturally acquired antimalarial antibodies. Most importantly, 10 of 11 Malian RH5-depleted IgGs and 9 of 10 Malian total IgGs did not display a detectable level of interference (i.e., a combination showing lower %GIA than either antibody alone) with any of the neutralizing RH5 antibodies tested (either mAbs or pAbs). This is in contrast to the interaction of AMA1-depleted Malian IgGs with neutralizing AMA1 antibodies. Assuming GIA activity correlates with *in vivo* outcome (as has been shown in *Aotus* monkey studies and human CHMI), this study suggests that antibody responses to an RH5 vaccine may combine independently (additively) with pre-existing GIA responses, giving a better outcome than vaccine alone and thus performing better than AMA1 vaccines in malaria-endemic areas. These results support further investigation of RH5 vaccines in target populations.

Limitations of study

The amount of each RH5-specific IgG pool was limited (each RH5-specific IgG pool was purified from 10–15 mL of serum); therefore, some experiments could not be performed (e.g., GIA at higher concentrations and IgG subclass determination) with these samples. We did not investigate the mechanism of synergy (with neutralizing RH5 mAbs) or antagonism (with VAC063 pooled IgGs), because only a minor proportion of Malian IgGs demonstrated such synergistic or antagonistic interactions, meaning we had a limited volume of material with which to investigate the mechanism. There is a risk of misclassification for the interactions (additive, synergistic, or antagonistic). As shown in the Supplemental information tables, there was a considerable level of inter-assay variation in %GIA values, whereas the variation between technical replicates on the same plate was relatively small, as measured by the percentage of the coefficient of variation (mean = 2.5, standard deviation = 2.5, n = 466 conditions). To assess the interaction effect, we opted to use DfB values instead of raw %GIA values, because the inter-assay

CellPress
OPEN ACCESS

variation in DfB was comparatively smaller (Figure S5; Pearson correlation coefficient r from two assays was >0.83, $p < 0.001$ for all three datasets). In addition, the cutoff value of ±10 to define synergy or antagonism is somewhat arbitrary. For example, if the average DfB value is −5, the antibody combination might have a weak but true antagonistic interaction, whereas it was labeled as an additive interaction in this study (drawing the conclusion that the two antibodies work independently). Nonetheless, we believe such a small effect would have a minimal biological impact. We performed all GIAs with a single strain of *P. falciparum*, 3D7. Although RH5 vaccination of animals[11,13,15] and humans[17] induced strain-transcending antibodies, as judged by GIA, further study is required to confirm whether the generally additive interactions observed in the current study hold true for other strains of parasites. Finally, it is difficult to predict complicated immune responses *in vivo* from these *in vitro* experiments. For example, pre-existing immunity in the target population may alter the quality and quantity of anti-RH5 antibodies induced by vaccination. Only clinical trials in the target population can prove or disprove the efficacy of vaccine.

STAR★METHODS

Detailed methods are provided in the online version of this paper and include the following:

- KEY RESOURCES TABLE
- RESOURCE AVAILABILITY
  - Lead contact
  - Materials availability
  - Data and code availability
- EXPERIMENTAL MODEL AND SUBJECT DETAILS
  - Malian cohort
  - RH5 human clinical trial (VAC063 trial)
- METHOD DETAILS
  - Recombinant proteins and monoclonal antibodies (mAbs)
  - Total and RH5-specific IgG purification
  - ELISA
  - GIA
- QUANTIFICATION AND STATISTICAL ANALYSIS
- ADDITIONAL RESOURCES

SUPPLEMENTAL INFORMATION

Supplemental information can be found online at https://doi.org/10.1016/j.xcrm.2021.100326.

ACKNOWLEDGMENTS

We thank all volunteers and the parents and guardians of children who participated in the Mali cohort study and VAC063 phase I/IIa trial; Seidina Diakite and Karim Traore for their contributions to the Mali cohort study; and the VAC063 clinical trial study team. The study was supported by the intramural program of the National Institute of Allergy and Infectious Diseases /NIH, and GIAs were partially supported by the U.S. Agency for International Development (USAID). D.G.W.A. held a UK Medical Research Council iCASE PhD Studentship (MR/K017632/1); S.J.D. is a Jenner investigator, a Lister Institute research prize fellow, and a Wellcome Trust senior fellow (106917/Z/15/Z). This work was also supported in part by the European Union's Horizon 2020 research and innovation programme under a grant agreement for OptiMalVax (733273). The VAC063 clinical trial was funded by the Office of Infectious Diseases, Bureau for Global Health, USAID, under the terms of the Malaria Vaccine Development Program (MVDP) (AID-OAA-C-15-00071), for which Leidos Inc. is the prime contractor. The opinions expressed herein are those of the authors and do not necessarily reflect the views of USAID. The funders had no role in study design, data collection and analysis, decision to publish, or preparation of the manuscript.

AUTHOR CONTRIBUTIONS

A.C.W., K.M., and C.A.L. designed the study; M.D., A.M.M., S.E.S., J.R.B., and S.J.D. coordinated and carried out the cohort and the clinical trial; D.P., D.G.W.A., and L.D.W.K. produced recombinant proteins and mAbs; A.C.W., A.D., and A.S.H. carried out laboratory procedures; A.C.W. and K.M. performed data analyses and prepared the initial draft of the manuscript; and S.J.D. and C.A.L. performed critical review and revision of the manuscript.

DECLARATION OF INTERESTS

D.G.W.A. and S.J.D. are named inventors on patent applications relating to RH5 vaccines and/or antibodies. A.M.M. has an immediate family member who is an inventor on patents relating to RH5 vaccines and/or antibodies. The authors declare no other competing interests.

Received: January 11, 2021
Revised: May 3, 2021
Accepted: June 4, 2021
Published: June 21, 2021

REFERENCES

1. World Health Organization (2020). World malaria report 2019 (World Health Organization).
2. Crosnier, C., Bustamante, L.Y., Bartholdson, S.J., Bei, A.K., Theron, M., Uchikawa, M., Mboup, S., Ndir, O., Kwiatkowski, D.P., Duraisingh, M.T., et al. (2011). Basigin is a receptor essential for erythrocyte invasion by *Plasmodium falciparum*. Nature *480*, 534–537.
3. Wong, W., Huang, R., Menant, S., Hong, C., Sandow, J.J., Birkinshaw, R.W., Healer, J., Hodder, A.N., Kanjee, U., Tonkin, C.J., et al. (2019). Structure of *Plasmodium falciparum* Rh5-CyRPA-Ripr invasion complex. Nature *565*, 118–121.
4. Volz, J.C., Yap, A., Sisquella, X., Thompson, J.K., Lim, N.T., Whitehead, L.W., Chen, L., Lampe, M., Tham, W.H., Wilson, D., et al. (2016). Essential role of the PfRh5/PfRipr/CyRPA complex during *Plasmodium falciparum* invasion of erythrocytes. Cell Host Microbe *20*, 60–71.
5. Tran, T.M., Ongoiba, A., Coursen, J., Crosnier, C., Diouf, A., Huang, C.Y., Li, S., Doumbo, S., Doumtabe, D., Kone, Y., et al. (2014). Naturally acquired antibodies specific for *Plasmodium falciparum* reticulocyte-binding protein homologue 5 inhibit parasite growth and predict protection from malaria. J. Infect. Dis. *209*, 789–798.
6. Richards, J.S., Arumugam, T.U., Reiling, L., Healer, J., Hodder, A.N., Fowkes, F.J., Cross, N., Langer, C., Takeo, S., Uboldi, A.D., et al. (2013). Identification and prioritization of merozoite antigens as targets of protective human immunity to *Plasmodium falciparum* malaria for vaccine and biomarker development. J. Immunol. *191*, 795–809.
7. Chiu, C.Y., Healer, J., Thompson, J.K., Chen, L., Kaul, A., Savergave, L., Raghuwanshi, A., Li Wai Suen, C.S., Siba, P.M., Schofield, L., et al. (2014). Association of antibodies to *Plasmodium falciparum* reticulocyte binding protein homolog 5 with protection from clinical malaria. Front. Microbiol. *5*, 314.
8. Weaver, R., Reiling, L., Feng, G., Drew, D.R., Mueller, I., Siba, P.M., Tsuboi, T., Richards, J.S., Fowkes, F.J.I., and Beeson, J.G. (2016). The association between naturally acquired IgG subclass specific antibodies to the

PfRH5 invasion complex and protection from *Plasmodium falciparum* malaria. Sci. Rep. *6*, 33094.

9. Douglas, A.D., Baldeviano, G.C., Lucas, C.M., Lugo-Roman, L.A., Crosnier, C., Bartholdson, S.J., Diouf, A., Miura, K., Lambert, L.E., Ventocilla, J.A., et al. (2015). A PfRH5-based vaccine is efficacious against heterologous strain blood-stage *Plasmodium falciparum* infection in *aotus* monkeys. Cell Host Microbe *17*, 130–139.
10. Douglas, A.D., Baldeviano, G.C., Jin, J., Miura, K., Diouf, A., Zenonos, Z.A., Ventocilla, J.A., Silk, S.E., Marshall, J.M., Alanine, D.G.W., et al. (2019). A defined mechanistic correlate of protection against *Plasmodium falciparum* malaria in non-human primates. Nat. Commun. *10*, 1953.
11. Douglas, A.D., Williams, A.R., Illingworth, J.J., Kamuyu, G., Biswas, S., Goodman, A.L., Wyllie, D.H., Crosnier, C., Miura, K., Wright, G.J., et al. (2011). The blood-stage malaria antigen PfRH5 is susceptible to vaccine-inducible cross-strain neutralizing antibody. Nat. Commun. *2*, 601.
12. Ord, R.L., Rodriguez, M., Yamasaki, T., Takeo, S., Tsuboi, T., and Lobo, C.A. (2012). Targeting sialic acid dependent and independent pathways of invasion in *Plasmodium falciparum*. PLoS ONE *7*, e30251.
13. Williams, A.R., Douglas, A.D., Miura, K., Illingworth, J.J., Choudhary, P., Murungi, L.M., Furze, J.M., Diouf, A., Miotto, O., Crosnier, C., et al. (2012). Enhancing blockade of *Plasmodium falciparum* erythrocyte invasion: assessing combinations of antibodies against PfRH5 and other merozoite antigens. PLoS Pathog. *8*, e1002991.
14. Bustamante, L.Y., Bartholdson, S.J., Crosnier, C., Campos, M.G., Wanaguru, M., Nguon, C., Kwiatkowski, D.P., Wright, G.J., and Rayner, J.C. (2013). A full-length recombinant *Plasmodium falciparum* PfRH5 protein induces inhibitory antibodies that are effective across common PfRH5 genetic variants. Vaccine *31*, 373–379.
15. Reddy, K.S., Pandey, A.K., Singh, H., Sahar, T., Emmanuel, A., Chitnis, C.E., Chauhan, V.S., and Gaur, D. (2014). Bacterially expressed full-length recombinant *Plasmodium falciparum* RH5 protein binds erythrocytes and elicits potent strain-transcending parasite-neutralizing antibodies. Infect. Immun. *82*, 152–164.
16. Healer, J., Wong, W., Thompson, J.K., He, W., Birkinshaw, R.W., Miura, K., Long, C.A., Soroka, V., Søgaard, T.M.M., Jørgensen, T., et al. (2019). Neutralising antibodies block the function of Rh5/Ripr/CyRPA complex during invasion of *Plasmodium falciparum* into human erythrocytes. Cell. Microbiol. *21*, e13030.
17. Payne, R.O., Silk, S.E., Elias, S.C., Miura, K., Diouf, A., Galaway, F., de Graaf, H., Brendish, N.J., Poulton, I.D., Griffiths, O.J., et al. (2017). Human vaccination against RH5 induces neutralizing antimalarial antibodies that inhibit RH5 invasion complex interactions. JCI Insight *2*, 96381.
18. Alanine, D.G.W., Quinkert, D., Kumarasingha, R., Mehmood, S., Donnellan, F.R., Minkah, N.K., Dadonaite, B., Diouf, A., Galaway, F., Silk, S.E., et al. (2019). Human antibodies that slow erythrocyte invasion potentiate malaria-neutralizing antibodies. Cell *178*, 216–228.e21.
19. Jin, J., Tarrant, R.D., Bolam, E.J., Angell-Manning, P., Soegaard, M., Pattinson, D.J., Dulal, P., Silk, S.E., Marshall, J.M., Dabbs, R.A., et al. (2018). Production, quality control, stability, and potency of cGMP-produced *Plasmodium falciparum* RH5.1 protein vaccine expressed in *Drosophila* S2 cells. NPJ Vaccines *3*, 32.
20. Minassian, A.M., Silk, S.E., Barrett, J.R., Nielsen, C.M., Miura, K., Diouf, A., Loos, C., Fallon, J.K., Michell, A.R., White, M.T., et al. (2021). Reduced blood-stage malaria growth and immune correlates in humans following RH5 vaccination. Med *2*, 1–19.
21. Miura, K. (2016). Progress and prospects for blood-stage malaria vaccines. Expert Rev. Vaccines *15*, 765–781.
22. Thera, M.A., Doumbo, O.K., Coulibaly, D., Laurens, M.B., Ouattara, A., Kone, A.K., Guindo, A.B., Traore, K., Traore, I., Kouriba, B., et al. (2011). A field trial to assess a blood-stage malaria vaccine. N. Engl. J. Med. *365*, 1004–1013.
23. Sagara, I., Dicko, A., Ellis, R.D., Fay, M.P., Diawara, S.I., Assadou, M.H., Sissoko, M.S., Kone, M., Diallo, A.I., Saye, R., et al. (2009). A randomized controlled phase 2 trial of the blood stage AMA1-C1/Alhydrogel malaria vaccine in children in Mali. Vaccine *27*, 3090–3098.
24. Ragotte, R.J., Higgins, M.K., and Draper, S.J. (2020). The RH5-CyRPA-Ripr complex as a malaria vaccine target. Trends Parasitol. *36*, 545–559.
25. Remarque, E.J., Faber, B.W., Kocken, C.H., and Thomas, A.W. (2008). Apical membrane antigen 1: a malaria vaccine candidate in review. Trends Parasitol. *24*, 74–84.
26. Miura, K., Zhou, H., Diouf, A., Tullo, G., Moretz, S.E., Aebig, J.A., Fay, M.P., Miller, L.H., Doumbo, O.K., Sagara, I., et al. (2011). Immunological responses against *Plasmodium falciparum* Apical Membrane Antigen 1 vaccines vary depending on the population immunized. Vaccine *29*, 2255–2261.
27. Miura, K., Zhou, H., Moretz, S.E., Diouf, A., Thera, M.A., Dolo, A., Doumbo, O., Malkin, E., Diemert, D., Miller, L.H., et al. (2008). Comparison of biological activity of human anti-apical membrane antigen-1 antibodies induced by natural infection and vaccination. J. Immunol. *181*, 8776–8783.
28. Miura, K., Perera, S., Brockley, S., Zhou, H., Aebig, J.A., Moretz, S.E., Miller, L.H., Doumbo, O.K., Sagara, I., Dicko, A., et al. (2011). Non-apical membrane antigen 1 (AMA1) IgGs from Malian children interfere with functional activity of AMA1 IgGs as judged by growth inhibition assay. PLoS ONE *6*, e20947.
29. Adomako-Ankomah, Y., Chenoweth, M.S., Durfee, K., Doumbia, S., Konate, D., Doumbouya, M., Keita, A.S., Nikolaeva, D., Tullo, G.S., Anderson, J.M., et al. (2017). High *Plasmodium falciparum* longitudinal prevalence is associated with high multiclonality and reduced clinical malaria risk in a seasonal transmission area of Mali. PLoS ONE *12*, e0170948.
30. Galaway, F., Drought, L.G., Fala, M., Cross, N., Kemp, A.C., Rayner, J.C., and Wright, G.J. (2017). P113 is a merozoite surface protein that binds the N terminus of *Plasmodium falciparum* RH5. Nat. Commun. *8*, 14333.
31. Osier, F.H., Mackinnon, M.J., Crosnier, C., Fegan, G., Kamuyu, G., Wanaguru, M., Ogada, E., McDade, B., Rayner, J.C., Wright, G.J., and Marsh, K. (2014). New antigens for a multicomponent blood-stage malaria vaccine. Sci. Transl. Med. *6*, 247ra102.
32. Partey, F.D., Castberg, F.C., Sarbah, E.W., Silk, S.E., Awandare, G.A., Draper, S.J., Opoku, N., Kweku, M., Ofori, M.F., Hviid, L., and Barfod, L. (2018). Kinetics of antibody responses to PfRH5-complex antigens in Ghanaian children with *Plasmodium falciparum* malaria. PLoS ONE *13*, e0198371.
33. Dreyer, A.M., Matile, H., Papastogiannidis, P., Kamber, J., Favuzza, P., Voss, T.S., Wittlin, S., and Pluschke, G. (2012). Passive immunoprotection of *Plasmodium falciparum*-infected mice designates the CyRPA as candidate malaria vaccine antigen. J. Immunol. *188*, 6225–6237.
34. Bustamante, L.Y., Powell, G.T., Lin, Y.C., Macklin, M.D., Cross, N., Kemp, A., Cawkill, P., Sanderson, T., Crosnier, C., Muller-Sienerth, N., et al. (2017). Synergistic malaria vaccine combinations identified by systematic antigen screening. Proc. Natl. Acad. Sci. USA *114*, 12045–12050.
35. Patel, S.D., Ahouidi, A.D., Bei, A.K., Dieye, T.N., Mboup, S., Harrison, S.C., and Duraisingh, M.T. (2013). *Plasmodium falciparum* merozoite surface antigen, PfRH5, elicits detectable levels of invasion-inhibiting antibodies in humans. J. Infect. Dis. *208*, 1679–1687.
36. Ord, R.L., Caldeira, J.C., Rodriguez, M., Noe, A., Chackerian, B., Peabody, D.S., Gutierrez, G., and Lobo, C.A. (2014). A malaria vaccine candidate based on an epitope of the *Plasmodium falciparum* RH5 protein. Malar. J. *13*, 326.
37. Chen, L., Xu, Y., Wong, W., Thompson, J.K., Healer, J., Goddard-Borger, E.D., Lawrence, M.C., and Cowman, A.F. (2017). Structural basis for inhibition of erythrocyte invasion by antibodies to *Plasmodium falciparum* protein CyRPA. eLife *6*, e21347.
38. Reddy, K.S., Amlabu, E., Pandey, A.K., Mitra, P., Chauhan, V.S., and Gaur, D. (2015). Multiprotein complex between the GPI-anchored CyRPA with PfRH5 and PfRipr is crucial for *Plasmodium falciparum* erythrocyte invasion. Proc. Natl. Acad. Sci. USA *112*, 1179–1184.

CellPress
OPEN ACCESS

39. Favuzza, P., Guffart, E., Tamborrini, M., Scherer, B., Dreyer, A.M., Rufer, A.C., Erny, J., Hoernschemeyer, J., Thoma, R., Schmid, G., et al. (2017). Structure of the malaria vaccine candidate antigen CyRPA and its complex with a parasite invasion inhibitory antibody. eLife *6*, e20383.

40. Illingworth, J.J., Alanine, D.G., Brown, R., Marshall, J.M., Bartlett, H.E., Silk, S.E., Labbé, G.M., Quinkert, D., Cho, J.S., Wendler, J.P., et al. (2019). Functional comparison of blood-stage *Plasmodium falciparum* malaria vaccine candidate antigens. Front. Immunol. *10*, 1254.

41. Azasi, Y., Gallagher, S.K., Diouf, A., Dabbs, R.A., Jin, J., Mian, S.Y., Narum, D.L., Long, C.A., Gaur, D., Draper, S.J., et al. (2020). Bliss' and Loewe's additive and synergistic effects in *Plasmodium falciparum* growth inhibition by AMA1-RON2L, RH5, RIPR and CyRPA antibody combinations. Sci. Rep. *10*, 11802.

42. Rijal, P., Elias, S.C., Machado, S.R., Xiao, J., Schimanski, L., O'Dowd, V., Baker, T., Barry, E., Mendelsohn, S.C., Cherry, C.J., et al. (2019). Therapeutic monoclonal antibodies for Ebola virus infection derived from vaccinated humans. Cell Rep. *27*, 172–186.e7.

43. Miura, K., Orcutt, A.C., Muratova, O.V., Miller, L.H., Saul, A., and Long, C.A. (2008). Development and characterization of a standardized ELISA including a reference serum on each plate to detect antibodies induced by experimental malaria vaccines. Vaccine *26*, 193–200.

44. Malkin, E.M., Diemert, D.J., McArthur, J.H., Perreault, J.R., Miles, A.P., Giersing, B.K., Mullen, G.E., Orcutt, A., Muratova, O., Awkal, M., et al. (2005). Phase 1 clinical trial of apical membrane antigen 1: an asexual blood-stage vaccine for *Plasmodium falciparum* malaria. Infect. Immun. *73*, 3677–3685.

45. Bliss, C.I. (1939). The toxicity of poisons applied jointly. Ann. Appl. Biol. *26*, 585–615.

STAR★METHODS

KEY RESOURCES TABLE

REAGENT or RESOURCE	SOURCE	IDENTIFIER
Antibodies		
R5.010, R5.011, R5.014, R5.004, R5.008 and R5.016	Simon J. Draper, Oxford University: Alanine et al.[18]	N/A
EBL040	Simon J. Draper, Oxford University: Rijal et al.[42]	N/A
Alkaline Phosphatase Labeled Goat anti-Human IgG (H+L)	Kirkegaard & Perry Labs	Cat # 075-1006
Biological samples		
Malian sera	Carole A Long, NIAID: Adomakko-Ankomah et al.[29]	N/A
VAC063 sera	Simon J. Draper, Oxford University, Clinicaltrials.gov: NCT02927145	N/A
Chemicals, peptides, and recombinant proteins		
RH5.1	Simon J. Draper, Oxford University: Jin et al.[19]	N/A
Full-length Ripr	Simon J. Draper, Oxford University: Alanine et al.[18]	N/A
Full-length CyRPA	Simon J. Draper, Oxford University: Alanine et al.[18]	N/A
RH5ΔNL	Simon J. Draper, Oxford University: Alanine et al.[18]	N/A
RH5Nt	Simon J. Draper, Oxford University: Galaway et al.[30]	N/A
NHS-activated Sepharose 4 Fast Flow	Cytiva	Cat# 17090601
Experimental models: Organisms/strains		
P. falciparum 3D7	Carole A Long, NIAID	N/A
P. falciparum FVO	Carole A Long, NIAID	N/A
Software and algorithms		
GraphPad Prism version 8	GraphPad (https://www.graphpad.com/)	N/A

RESOURCE AVAILABILITY

Lead contact

Further information and requests for resources and reagents should be directed to and will be fulfilled by the Lead Contact, Kazutoyo Miura (kmiura@niaid.nih.gov).

Materials availability

There are restrictions to the availability of the human sera described in this manuscript due to ethical approvals for the studies. Requests directed to the Lead Contact will be considered on an individual basis.

Data and code availability

The published article includes all datasets generated or analyzed during this study, and no code was generated in this study.

EXPERIMENTAL MODEL AND SUBJECT DETAILS

Malian cohort

Details of the study site and cohort have been described previously.[29] Briefly, the study was conducted with 500 individuals aged 1-65 living in the village of Kenieroba, Mali, an area of high malaria transmission with a rainy season from June to December. Blood samples were collected from participants every other week from June 2013 to May 2014. Detection of infection with *P. falciparum* was performed using a species-specific nested PCR, as described.[29] If participants presented with malaria symptoms during a scheduled visit or by self-referral to the health center, parasitemia was measured by microscopy, as described.[29] A malaria case was defined as an axillary temperature > 37.5°C and asexual parasite density > 5000/μL.

From 405 volunteers, 2-5 mL of serum were collected at the peak of the malaria transmission season (October 2013) and used for the current study. This study received approval from the Institutional Review Board of the National Institute of Allergy and Infectious Diseases and the Ethics Committee of the Faculty of Medicine, Pharmacy, and Odontostomatology, University of Bamako. Written

informed consent was obtained from study participants or the parents or guardians of children aged < 18 years, and the trial is registered at Clinicaltrials.gov: NCT01829737.

RH5 human clinical trial (VAC063 trial)

The details of the RH5.1/AS01$_B$ vaccine trial (VAC063 trial) were described.[20] In brief, the VAC063 trial was an open-label, non-randomized dose escalation Phase I/IIa trial using the RH5.1 protein[19] (full-length recombinant RH5 protein based on the 3D7 sequence) formulated in GSK's adjuvant system AS01$_B$. Healthy malaria-naive UK adults from 18 to 50 years old were immunized with 2, 10 or 50 μg of RH5.1 protein on days 0, 28 and 56, or days 0, 28 and 182. The trial was registered on Clinicaltrials.gov: NCT02927145, received ethical approval from the UK NHS Research Ethics Service (Oxfordshire Research Ethics Committee A, Ref 16/SC/0345), and was approved by the UK Medicines and Healthcare Products Regulatory Agency (Ref 21584/0362/001-0001). Written informed consent was obtained from study participants. For this study, 11 total IgG pools were made from the purified total IgGs of 59 vaccinees based on their RH5 antibody levels and available volumes, irrespective of immunization group or study day (samples used ranged from day 70 to 366 from Group 1, 2 or 6). Each pool consisted of 4 to 25 individual total IgGs.

METHOD DETAILS

Recombinant proteins and monoclonal antibodies (mAbs)

RH5.1 (amino acid, aa E26 to Q526),[19] full-length Ripr (aa D21 to N1086),[18] full-length CyRPA (aa D29 to E362),[18] RH5ΔNL (aa K140 to K247 and N297 to Q526),[18] and RH5Nt (aa F25 to K140)[30] were produced and purified as described previously. In brief, all constructs were made based on the 3D7 sequence of *P. falciparum* with mutations to delete N-linked glycosylation using *Drosophila* S2 (RH5.1, Ripr, RH5ΔNL) or Expi293F HEK (CyRPA, RH5Nt) expression systems. Recombinant proteins were purified from the cell supernatant using Capture Select C-tag (RH5.1, RH5ΔNL RIPR, CyRPA) or immobilized metal ion (RH5Nt) affinity chromatography, followed by size exclusion chromatography (SEC) into TBS (20 mM Tris, 150 mM NaCl) pH 7.4. The RH5 complex was reconstituted *in vitro* by incubating equimolar concentrations of RH5.1, CyRPA and Ripr proteins at 4°C for 1h before purifying by SEC. (Pulido D et al., in preparation).

The human RH5 mAbs used in the combination GIA were all cloned as human IgG1 and isolated from vaccinated participants in the VAC057 trial, in which they were immunized with replication-deficient chimpanzee adenovirus serotype 63 (ChAd63) and the attenuated orthopoxvirus modified vaccinia virus Ankara (MVA), both encoding full-length RH5.[17] Detailed descriptions of the mAbs have been reported previously.[18] Several non-neutralizing but potentiating RH5 mAbs (R5.010, R5.011, and R5.014) as well as neutralizing RH5 mAbs (R5.004, R5.008, and R5.016) were utilized in this study. As a negative control, a human Ebola virus mAb (EBL040)[42] was used.

Total and RH5-specific IgG purification

Using 138 individual Malian serum samples, 23 serum pools were generated (each pool consisted of 5 to 12 individual sera), and total IgGs were purified using Protein G columns. RH5-specific IgGs were purified from the total IgG pools by RH5 affinity purification using columns with RH5.1-coated beads. The flow-through fraction was also collected and represents RH5-depleted IgG. The methods for purifying RH5-specific and RH5-depleted IgGs were described previously.[27] RH5-specific IgGs could only be purified from eight of the 23 pooled total IgGs (RH5 antibody levels in the remaining 15 total IgG samples were too low for successful purification of RH5-specific IgGs). Similarly, RH5-specific IgGs were affinity purified from 8 (out of 11) pooled VAC063 total IgGs. Additional non-pooled total IgGs were also prepared from 24 individual Malian sera. As a negative control, total IgGs were purified from the serum of malaria-naive U.S. adults purchased from Interstate Blood Bank (Memphis, TN). All total, RH5-specific, and RH5-depleted IgGs were heat inactivated, pre-adsorbed with human erythrocytes, dialyzed against RPMI 1640, and sterilized with a 0.22 μM filter for use in GIA.

ELISA

As a preliminary ELISA screen for the Malian sera, the 405 serum samples were tested at a 1:200 dilution against RH5.1, Ripr and CyRPA proteins to identify seropositive samples. The OD cut-off for positivity was defined as any OD above the mean + 2 standard deviations of 12 malaria-naive U.S. sera. ELISA units (EU) against RH5.1 were determined for all seropositive serum samples using the standardized ELISA methodology as described previously[43] with a small modification: each sample was tested in duplicate, not triplicate.

403 of the 405 Malian sera were also tested by ELISA against *Plasmodium falciparum* FVO blood-stage parasite lysate. Two serum samples were not tested due to limited volume. Infected erythrocytes (majority late stage trophozoites) were lysed by saponin treatment and then sonicated in an ice bath. ELISA plates were coated with the lysate at approximately 5×10^4 parasites/well, and the standardized ELISA was performed as described above.

RH5.1 titers in total, RH5-specific and RH5-depleted IgGs were measured using the standardized ELISA, as described above. RH5-specific antibodies were also tested for binding to two truncated RH5 proteins (RH5ΔNL and RH5Nt) as well as the recombinant RH5 complex (RCR). Each sample was diluted to target an OD of 1.0 against RH5.1 and tested against both RH5.1 and RH5ΔNL, RH5Nt or RCR on the same plate. The results are reported as an OD ratio between RH5ΔNL and RH5.1, RH5Nt and RH5.1, or RCR and RH5.1.

An avidity ELISA was also performed using the RH5-specific IgGs. Each sample was diluted to target an OD of approximately 2.0 against RH5.1 protein, and the ELISA was run as usual with an added 20-minute incubation with urea in 1X TBS (urea concentrations ranging from 1 to 6 M) following the primary antibody incubation. The concentration of urea resulting in a 20% reduction in EU (IC_{20}) was calculated using linear regression.

GIA

GIAs assessing the ability of antibody samples to inhibit parasite growth were performed with the 3D7 clone of *P. falciparum* as described previously.[44] For the GIA with single IgGs, the samples were diluted in RPMI to achieve the indicated concentration in RH5.1 EU or mg/ml. The total and RH5-specific IgGs from the VAC063 trial were tested at serial dilutions.

Mixtures of antibodies were assessed in combination GIAs. For the combination GIAs, the RH5-specific antibodies from Malian total IgGs were tested at the highest concentration possible. Malian pooled total IgGs, RH5-depleted IgGs and individual total IgGs were tested at 10 mg/ml unless they showed > 50% inhibition at this concentration, in which case they were tested at 2.5 mg/ml, or at 1.25 mg/ml if they still showed > 50% inhibition at 2.5 mg/ml. The pooled VAC063 total IgGs were tested at 1 mg/mL (mixture with RH5 mAbs), 0.625 mg/mL (with Malian RH5-depleted IgGs, Figures 5A and 5B), or 1.25 mg/mL (with Malian total IgGs, Figure 5D), while the VAC063 RH5-specific IgGs were diluted to target ~60% inhibition in GIA (%GIA). Three non-neutralizing, but potentiating, RH5 mAbs (R5.010, R5.011, and R5.014) were tested at 300 μg/ml, as described previously.[18] Three neutralizing RH5 mAbs (R5.004, R5.008 and R5.016) were tested at 50, 125 and 19 μg/ml to target ~50%–60%GIA. A human Ebola virus mAb was used as the negative control mAb, and a U.S. total IgG was used as the negative control total IgG. The details of mixture conditions and the original %GIA value for each condition are shown in Tables S1, S2, S3, S4, and S5. Figure 4A shows GIA results from a single assay, and Figures 4B and 5 show average results from two independent assays.

QUANTIFICATION AND STATISTICAL ANALYSIS

Predicted % inhibition values for antibody combinations were calculated using the formula for Bliss additivity, originally developed to assess the toxicity of poisons in combination.[45] The following formula, specific for antibody combinations in GIA, was used in the current study:

$$\%GIA_{[A+B]} = \left[1 - \left(1 - \frac{\%GIA_A}{100}\right) * \left(1 - \frac{\%GIA_B}{100}\right)\right] * 100$$

where $\%GIA_{[A+B]}$ is the %GIA of a combination of antibody A and antibody B, $\%GIA_A$ is the %GIA when antibody A is tested alone, and $\%GIA_B$ is antibody B alone. The difference between Bliss predicted %GIA and the observed %GIA in the mixture is denoted as "Delta from Bliss (DfB)" in this manuscript. We define additive interactions as those that give observed %GIA values within 10% on either side of the value predicted by Bliss (i.e., DfB is $\pm$ 10). If the observed %GIA is 10% or more greater than the value predicted by Bliss additivity (i.e., DfB is $\geq$ 10), the interaction is considered "synergistic." If the observed %GIA is 10% or more less than predicted (i.e., DfB is ≤ -10), the interaction is considered "antagonistic." The "antagonistic" interaction is further categorized into "sub-additive" and "interference" interactions. The former means the %GIA in the antibody combination is higher than the %GIA of either antibody alone, and the latter denotes the combination shows lower %GIA than either antibody alone. Unless otherwise mentioned, average DfB values from two independent assays were utilized to judge the "additive," "synergistic" or "antagonistic" ("sub-additive" and "interference") interactions.

To determine the impact of age category on seropositivity among the individual Malian sera, a chi-square test for trend was used. A Spearman rank test was used for assessing correlations, a Fisher's exact test for comparing proportions of samples between two groups, and a Mann-Whitney test for comparing means of two groups. All data analyses were performed using GraphPad Prism version 8 (GraphPad Software Inc.).

ADDITIONAL RESOURCES

The Mali and UK trials are registered as Clinicaltrials.gov: NCT01829737 and NCT02927145, respectively.

Cell Reports Medicine, Volume *2*

Supplemental information

Antibodies from malaria-exposed Malians generally interact additively or synergistically with human vaccine-induced RH5 antibodies

Alexandra C. Willcox, Alex S. Huber, Ababacar Diouf, Jordan R. Barrett, Sarah E. Silk, David Pulido, Lloyd D.W. King, Daniel G.W. Alanine, Angela M. Minassian, Mahamadou Diakite, Simon J. Draper, Carole A. Long, and Kazutoyo Miura

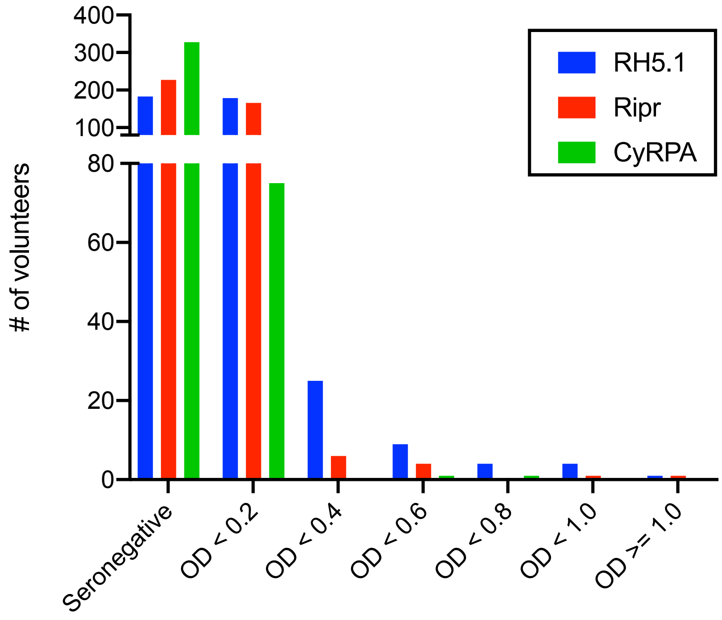

Figure S1: Antibody responses against RH5.1, Ripr and CyRPA. Related to Figure 1. Malian serum samples (n=405) and U.S. malaria-naive sera (n=12) were tested at 1:200 dilutions against the three proteins. After subtraction of background signal (i.e., average O.D. of the 12 U.S. sera), the Malian samples were categorized based on O.D. value.

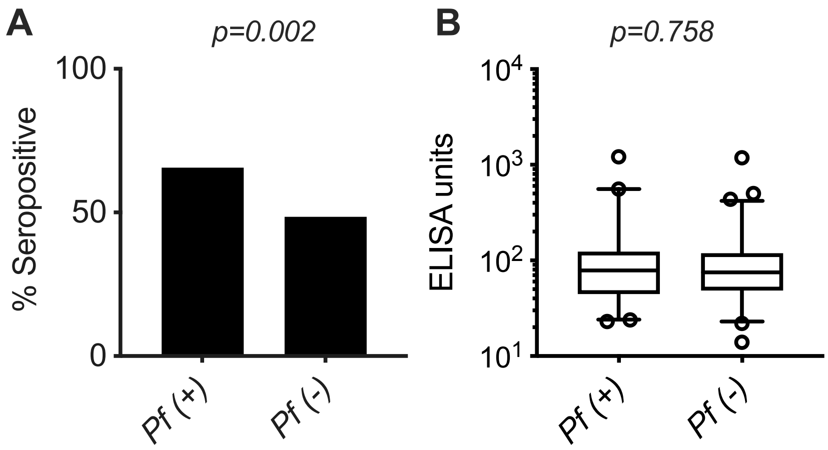

Figure S2: Association between *P. falciparum* infection at the time of sample collection and seropositivity for RH5.1. Related to Figure 1. Out of the 405 volunteers, 391 individuals had *P. falciparum* (Pf) infection status determined by PCR at the time of serum collection; 125 were Pf positive and 266 were Pf negative. (A) The proportion (%) of RH5.1 seropositive participants in each Pf positive or negative category is shown with a Fisher exact p value. (B) The distribution of ELISA units against RH5.1 in seropositive only participants with and without Pf infection (i.e. excluding seronegative participants; n=82 for Pf(+) and n=129 for Pf(-)). A box plot of the data with 95th percentile error bars is shown with the Mann-Whitney p-value.

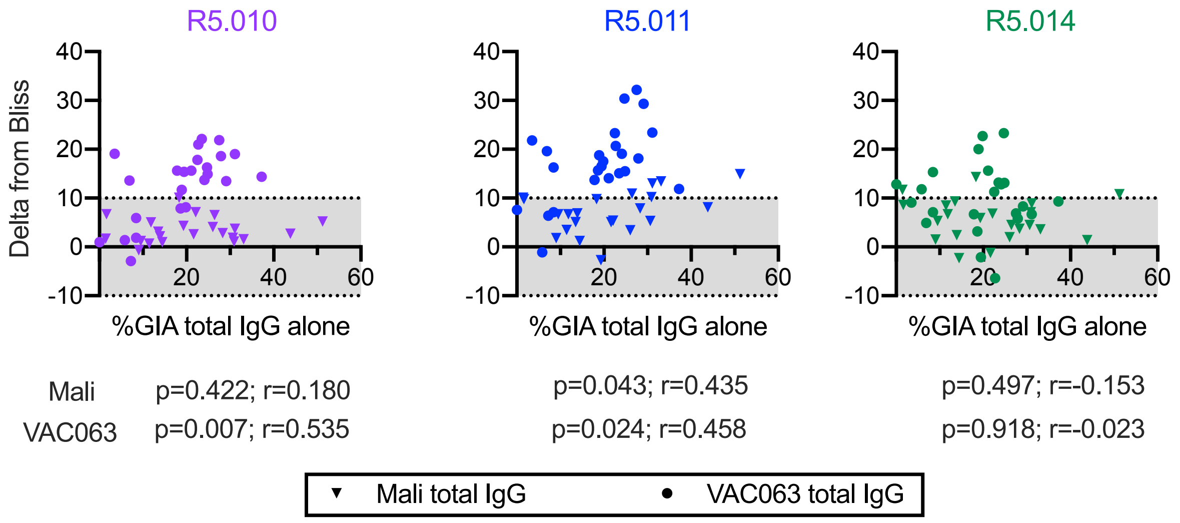

Figure S3: Initial screening data for combination GIA with potentiating RH5 mAbs. Related to Figure 4. Twenty-two Malian individual total IgGs and 24 VAC063 individual total IgGs were independently mixed with the three potentiating mAbs in GIA. The %GIA of total IgG alone is plotted against the Delta from Bliss value for combinations with each RH5 mAb. The Spearman rank test p-value and correlation coefficient r for each group of total IgGs against each mAb is shown. Within 10 Delta from Bliss results (gray areas) were considered additive (no potentiation).

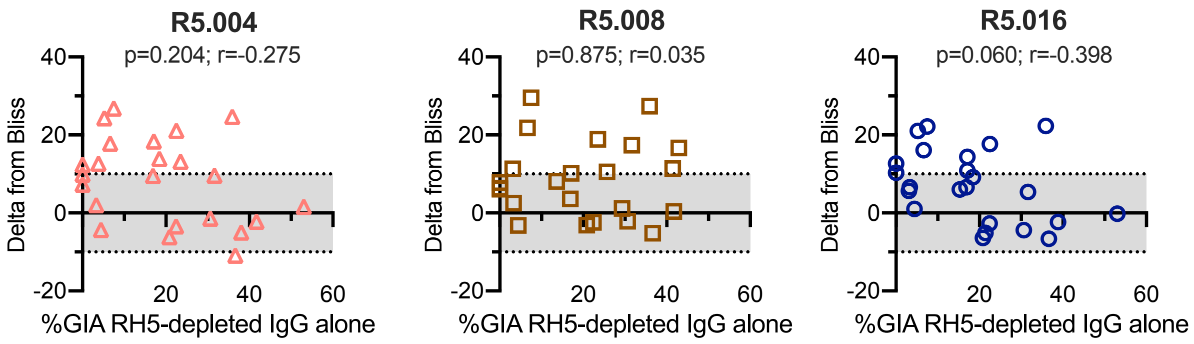

Figure S4: Association between GIA activity of Malian RH5-depleted IgG alone and Delta from Bliss value for combinations with RH5 mAbs. Related to Figure 5. The %GIA of each Malian RH5-depleted IgG alone is plotted against the Delta from Bliss value for combinations with each RH5 mAb, with Spearman rank test p-values and correlation coefficient r. Within 10 Delta from Bliss results (gray areas) were considered additive.

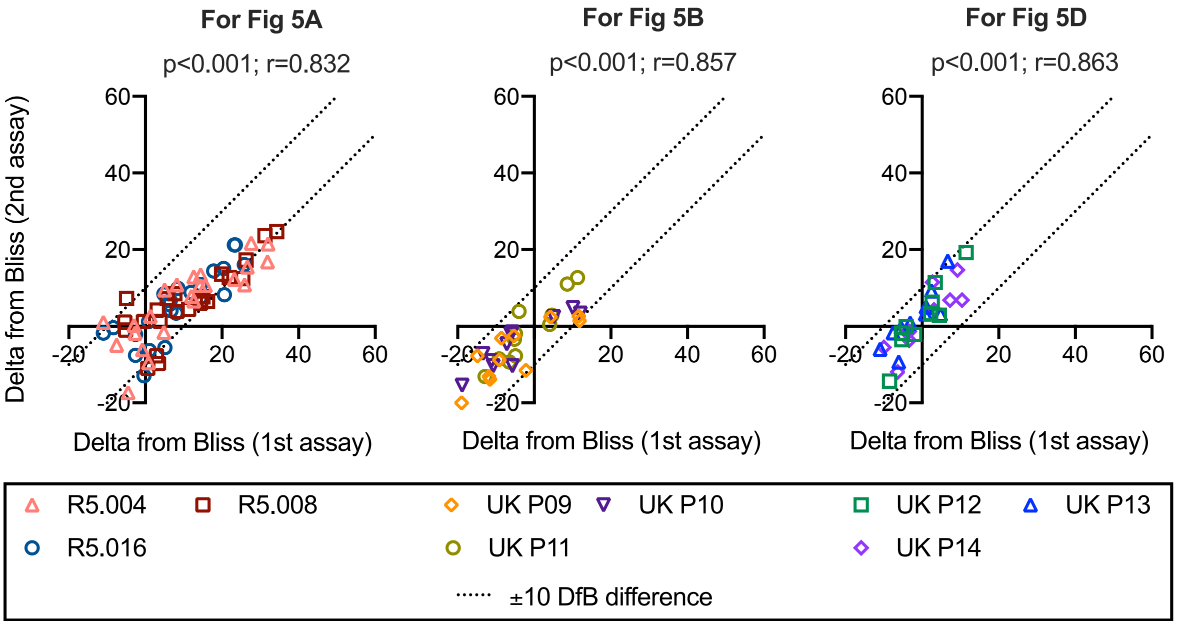

Figure S5: Inter-assay variability in Delta from Bliss value. Related to Figure 5. Independent combinations of Malian RH5-depleted IgGs and three RH5 mAbs (Fig 5A), combinations of Malian RH5-depleted IgGs and VAC063 pooled total IgGs (Fig 5B), or combinations of Malian total IgGs and VAC063 pooled total IgGs (Fig 5D) were assessed in two independent GIAs. The Delta from Bliss (DfB) value determined in the first assay was plotted against that from the second assay. The Pearson test p-value and correlation coefficient r for each data set is shown.
